# Pattern integration and differentiation: Dual process model of episodic memory

**DOI:** 10.1162/imag_a_00433

**Published:** 2025-01-09

**Authors:** Hallvard Røe Evensmoen, Lars M. Rimol, Henning Stople Rise, Tor Ivar Hansen, Hamed Nili, Anderson M. Winkler, Asta Håberg

**Affiliations:** Department of Neuromedicine and Movement Science, Norwegian University of Science and Technology (NTNU), Trondheim, Norway; MiDT National Research Center, Center for Innovation, Medical Equipment, and Technology, St. Olav’s Hospital, Trondheim University Hospital, Trondheim, Norway; Department of Psychology, Norwegian University of Science and Technology (NTNU), Trondheim, Norway; Department of Experimental Psychology, University of Oxford, Oxford, United Kingdom; Department of Human Genetics, University of Texas Rio Grande Valley, Brownsville, TX, United States

**Keywords:** sequence learning, temporal scaling, temporal distortions, pattern separation, medial temporal lobe, event boundaries

## Abstract

The role of precise timing in episodic memory remains obscure. We showed 139 participants episodes consisting of objects, and tested subsequent memory for the precise timing and order of the objects and episodes. Temporal compression of the episode enhanced memory for relative but not absolute timing of the objects’ presentation and their order. Conversely, temporal expansion between neighboring episodes was associated with successful memory for episode order. fMRI in 36 participants revealed that temporal compression of the episode was associated with more similar activation patterns within episodes in several brain regions including the posterior hippocampus. However, the activation pattern in the anterior hippocampus and other brain regions became more unique with temporal expansion between neighboring episodes. We propose that human episodic memory relies on two fundamentally opposite processes: pattern integration helps strengthen the relationship between the items that make up an episode and pattern differentiation keeps different episodes apart.

## 1 Introduction

A defining feature of episodic memory is an accurate representation of the order in which a series of items occurred (Clewett et al., 2019;Cohn-Sheehy & Ranganath, 2017;DuBrow & Davachi, 2017;Friedman, 1993;Lehn et al., 2009;Tulving, 2002) (Fig. 2). However, it is not known to what extent the human brain utilizes precise timing information when encoding a series of items (Clewett et al., 2019;Friedman, 1993;Teki et al., 2017). It is also unknown whether information about precise timing persists across multiple items or after a significant time delay. However, it is well documented that human perception of time tends to be distorted (Clewett et al., 2019). For instance, the temporal interval between two items is expanded across episodic or event boundaries as defined by changes in context or internal task sets (Brunec et al., 2018;Clewett et al., 2020;Faber & Gennari, 2017;Wang & Egner, 2022;Zacks, 2020). Furthermore, the memory for order across episodic boundaries is reported to be both reduced (DuBrow & Davachi, 2013,^2014^;Heusser et al., 2018;Horner et al., 2016;Pu et al., 2022) and enhanced (Wen & Egner, 2022). These findings suggest that temporal expansion across episodic boundaries is related to temporal order in episodic memory. However, whether the association between temporal expansion across episodic boundaries and order results from temporal compression within episodes and/or temporal expansion between episodes remains unknown. We hypothesize that in memory, temporal compression of episodes is associated with more accurate representation of item order within episodes, while temporal expansion between episodes provides a precise representation of episode order.

Two potential underlying neural mechanisms for temporal compression and expansion in memory are pattern integration and pattern differentiation, respectively. Pattern integration in the form of integration of various traces of activity in a population of neurons is considered necessary for storing an episode in long-term memory (Baldassano et al., 2017;Brunec et al., 2020;Clewett et al., 2019;DuBrow & Davachi, 2017). In support of this theory, hippocampal activation patterns become more similar or integrated when two stimuli are from the same sequence (Hsieh et al., 2014), always appear successively (Schapiro et al., 2012), are from the same temporal community (Schapiro et al., 2016), are closer in time (Kyle et al., 2015), are perceived as being closer in time (Deuker et al., 2016;Ezzyat & Davachi, 2014;Nielson et al., 2015), and with more accurate temporal order memory (DuBrow & Davachi, 2014). Successful memory is also considered to involve “pattern differentiation” of adjacent episodes, which can enhance the uniqueness of each episode (DuBrow & Davachi, 2017). Supporting this, episode-specific activation patterns in the lateral prefrontal cortex were more unique when episode order was more accurately recalled (Jenkins & Ranganath, 2010). Further, hippocampal activation patterns have been shown to separate individual episodes, based on the order and precise timing of the stimuli within the episodes (Barnett et al., 2014;Lee et al., 2020;Thavabalasingam et al., 2018,^2019^). Thus, pattern integration and pattern differentiation in the hippocampus and possibly lateral prefrontal cortex appear to support temporal aspects of episodic memory. However, it is unclear “[w]hether there are specific principles or guidelines that determine when memories become integrated or separated” (Clewett et al., 2019), and providing an understanding of these principles is “critical for establishing a comprehensive model of temporal memory” (DuBrow & Davachi, 2017).

**Fig. 1.**
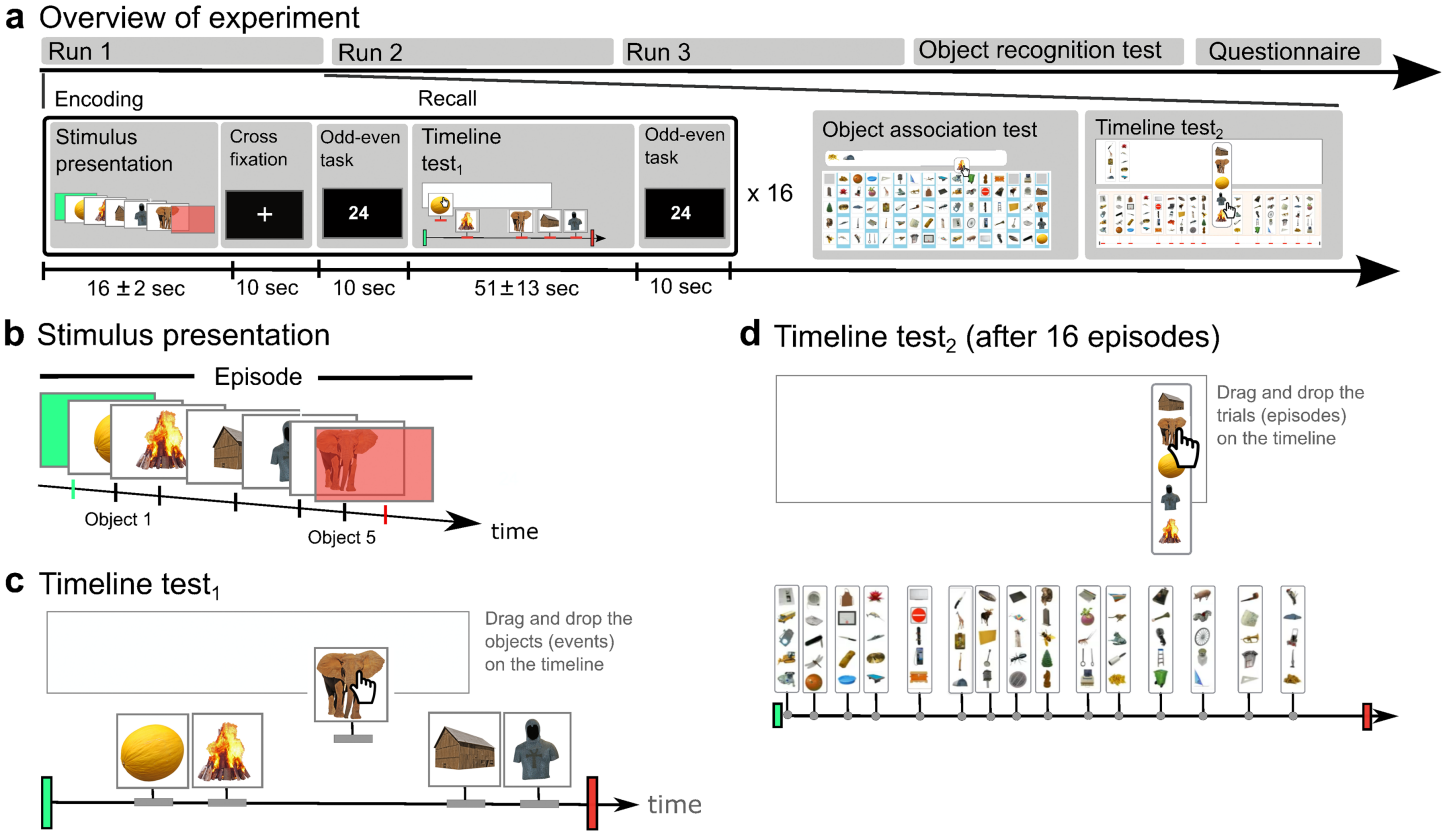
Experimental design. (a) Outline of the experiment with the top row showing the total number of runs and tests performed (see alsoSupplementary Fig. S1), and the bottom row showing the experimental design within a single run. The encoding consisted of the presentation of five objects in an episode, followed by a cross-fixation period in which further memorization took place, and, subsequently, an odd–even judgment task for consolidation. There were 16 unique episodes presented within each run. (b) Each episode consisted of five objects. A green screen marked the start of the episode and a red screen marked the end (“episode boundaries”). (c) After the odd–even judgment, the participants completed the Timeline test_1_, in which the participants were asked to position the objects he/she had seen as accurately as possible with regard to timing and order by dragging and dropping the objects onto an empty timeline representing the entire episode. (d) At the end of a run, after being exposed to 16 episodes, the participant completed Timeline test_2_and the object association test (see alsoSupplementary Fig. S1a). In Timeline test_2_, the participants were asked to position the episodes as accurately as possible with regard to timing and order, by dragging-and-dropping the episodes onto an empty timeline representing the entire run.

Our aim was to investigate the role of temporal compression and expansion in episodic memory, as well as the underlying neuronal mechanisms of neuronal correlates of pattern integration and pattern differentiation. We exposed human participants to a series of “episodes”, with each episode consisting of a sequence of objects appearing on a screen (Fig. 1). The participants were asked to memorize (encode) the timing and order of the objects in all episodes for later memory tests. Crucially, the experimental design (Figs. 1and2) allowed us to separate encoding of precise timing of object presentation and the order in which the objects were presented. Furthermore, the design preserved the “temporal pattern” of object and episode presentations, as defined by the relative time intervals between objects and episodes, respectively. This feature is important as humans tend to distort time. From the participants’ responses, we evaluated the role of relative timing, temporal compression, or expansion (scaling), as well as translation (i.e., distortion relative to the episode or run boundaries) within and between episodes. We asked the following questions: (1) Does precise timing play a role in human episodic memory within and between episodes? (2) Does pattern integration through temporal compression and pattern differentiation through temporal expansion take place within and between episodes, respectively? (3) Are pattern integration and differentiation at the behavioral level supported by a similar distinction in brain activation patterns in regions such as the hippocampus and lateral prefrontal cortex?

**Fig. 2.**
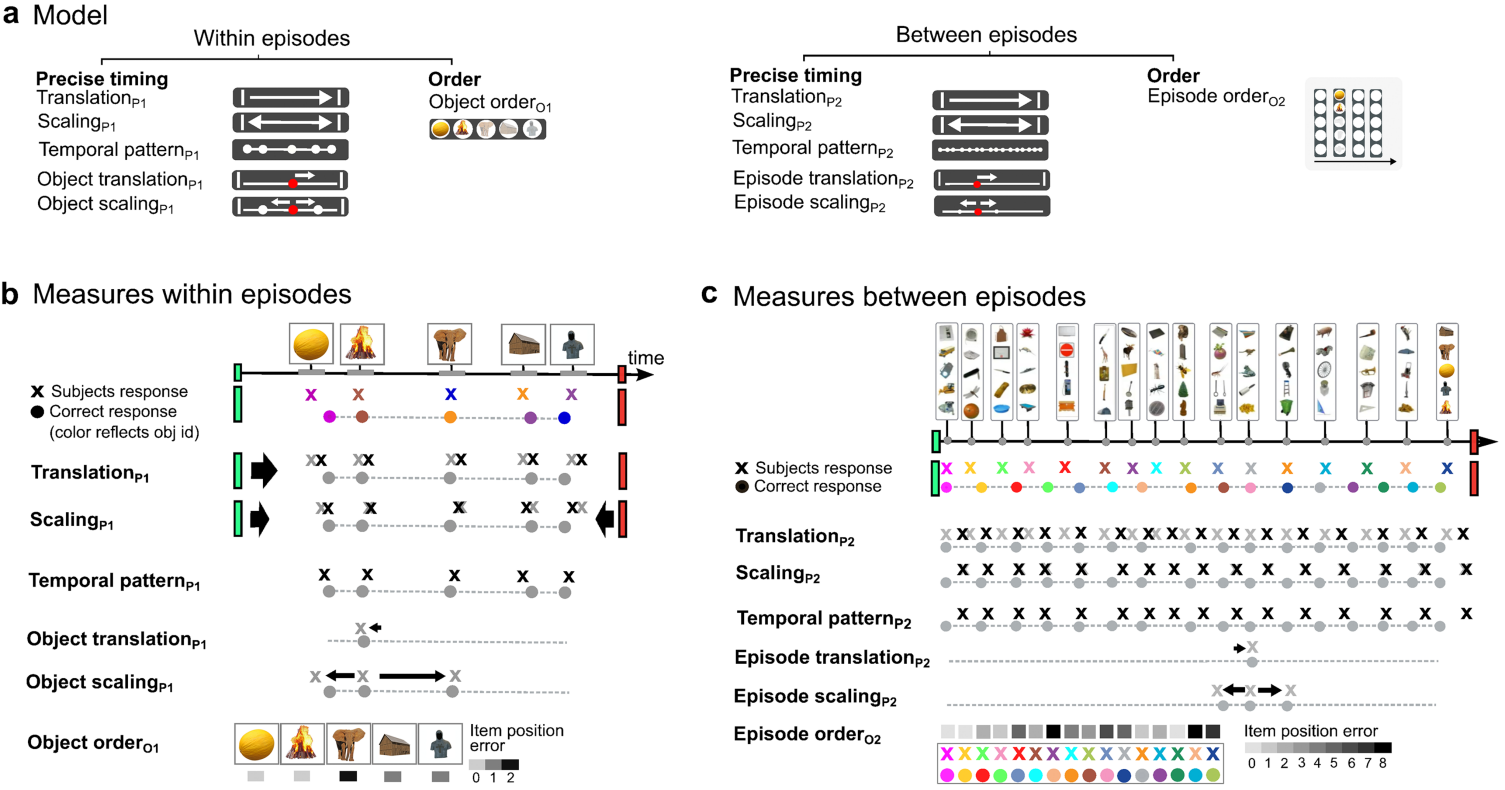
Temporal measures. (a) The participants’ representation of time in memory was divided into*precise timing*, which includes the exact temporal interval between specific objects and episodes, and*order*which represents the serial order of the objects and episodes. (b) From Timeline test_1_, precise timing and order measures within episodes were obtained. The top row shows the actual responses from one participant, with colors indicating object identity and the green and red screen indicating the temporal boundaries of the episode. Precise timing within episodes reflects the degree to which the participant reconstructed the exact timing of the objects, that is, the timing of the object relative to each other (Temporal patternP1), as well as timing relative to the temporal boundaries of the episode (Translation_P1_), and temporal expansion or compression of the time between the object presentations (ScalingP1) (see Methods for a detailed explanation). For individual objects, we estimated their precise position on the timeline (*Object translation_P1_*), as well as whether the time between one object and the object immediately preceding and/or following it in an episode was compressed or expanded (*Episode scaling_P1_*).*Object orderO1*reflects how far off the participants’ object order was from the correct object order, that is, how far off the sequence position of each of the objects in memory was from the correct sequence position. (c) From Timeline test_2_, the participant’s memory for the timing between and order of episodes was assessed. Precise timing between episodes reflects whether the participant’s response preserved the timing of the episodes in a run relative to each other (*Temporal patternP2*) and relative to the start and end of the run (*Translation_P1_*), as well as the exact temporal intervals between the episodes (ScalingP2). For individual episodes, we estimated their precise position on the timeline (Episode translation_P2_), as well as whether the intervals to neighboring episodes were compressed or expanded (Episode scalingP2).*Episode orderO2*was estimated by calculating, for each episode, how far off the sequence position of the episode in memory was relative to the correct sequence position.

## 2 Methods

### 2.1 Participants

One hundred and thirty-nine participants (age: 18–41 years, mean 24 years) with no history of neurological disorders, head trauma, previous or current DSM-IV axis I diagnosis of psychiatric illness, including substance abuse, were recruited to the study. All were right handed, as ascertained with the Edinburgh Handedness Inventory (Oldfield, 1971), with a mean score of 87.3 ± 14.4%. Two participants did not fill in the questionnaire related to the strategies used to encode and retrieve the object and episode sequences, and were, therefore, excluded from the mixed linear model behavioral analyses. Ninety-seven participants were male. All participants provided written informed consent prior to participation. The study was approved by the Regional Committee for Medical Research Ethics in central Norway. Thirty-six of the 139 participants completed the fMRI acquisition (22 males), but 1 male was excluded from the fMRI analysis because of excessive motion (average frame displacement >0.3 mm).

### 2.2 Paradigm

We exposed the 139 participants to 48 unique sequences (“episodes”) of 5 unrelated objects (Fig. 1). The participants’ memory for the episodes were then tested and several temporal and nontemporal measures extracted, including the temporal pattern and order of the objects and the episodes (Table 1;Fig. 2). The large number of episodes made it possible to get a robust estimate of the encoding of the different temporal and nontemporal aspects of the episodes and the relationship between encoding of the temporal and nontemporal aspects. For each episode, there were five periods; stimulus presentation that involved a random sequence of five unique objects (16.0 ± 2.0 seconds), cross-fixation (10 seconds), an odd–even task (10 seconds), Timeline test_1_(50.5 ± 13.0 seconds), and an odd–even task (10 seconds).

**Table 1.** Overview of the temporal and object measures.

Level	Memory type		Variable name	Explanation
Within episodes	Precise timing	Multiple temporal positions	Translation _P1_ 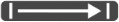	How accurately the participants placed the objects on the timeline relative to the boundaries of the episode, i.e., the distance between the geometric center of the objects positioned by the participant and the geometric center of the correct temporal presentation of the objects.
			Scaling _P1_ 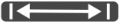	Whether the time between the objects positioned on the timeline by the participant was compressed or expanded compared with the original/correct temporal intervals in an episode.
			Scaling _P1_^Compr^	The degree to which the participant temporally compressed the time between the objects on the timeline compared with the original/correct temporal presentation in an episode.
			Scaling _P1_^Exp^	The degree to which the participant temporally expanded the time between the objects on the timeline compared with the original/correct positioning in the episode.
			Temporal pattern _P1_ 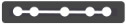	How accurately the participant placed the objects relative to each other, i.e., the deviation between the recalled and original temporal pattern of the objects. This measure allows for both translation and scaling of the temporal pattern, and as such only considers the relative timing of objects positioned on the timeline.
		Singular temporal positions	Object translation _P1_ 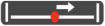	Temporal deviation between the participant’s positioning of one object and the original/correct object presentation within an episode.
			Object scaling _P1_ 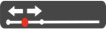	A scaling factor that indicates whether the participant compressed or expanded the time between an object and the object before and/or after, compared with the original/correct temporal intervals.
	Order		Object order _O1_ 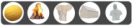	How accurately the participant remembered the order or sequence positions of the objects in an episode, calculated as the distance between the sequence position of the objects and the original/correct sequence positions.
Between episodes	Precise timing	Multiple temporal positions	Translation _P2_ 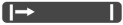	How accurately the participant placed the episodes on the timeline relative to the start and stop of the run, i.e., the difference between the geometric center of the episodes positioned by the participant and the geometric center of the correct temporal presentation of the episodes
			Scaling _P2_ 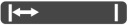	Whether the participant compressed or expanded the time between episodes positioned along the timeline, compared with the original/correct presentation of the episodes.
			Scaling _P2_^Compr^	The degree to which the participant temporally compressed the time between the episodes on the timeline compared with the original episode presentation.
			Scaling _P2_^Exp^	The degree to which the participant temporally expanded the time between the episodes on the timeline compared with the original episode presentation.
			Temporal pattern _P2_ 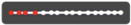	How accurately the participant remembered the relative time between the episodes within a run, allowing for both translation and scaling, and as such only considering the relative timing of episodes positioned compared with the original temporal presentation of the episodes.
		Individual temporal positions	Episode translation _P2_ 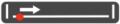	Temporal deviation between the participants positioning of an individual episode and the correct position.
			Episode scaling _P2_ 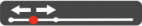	A scaling factor that indicates whether the participant compressed or expanded the time between an episode and the episode before and/or after, compared with the original/correct temporal intervals.
	Order		Episode order _O2_ 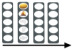	How accurately the participant remembered the order or sequence position of the different episodes in a run, calculated as the number of positions between the episode sequence position on the timeline and the original/correct sequence position.
	Nontemporal		Object recognition	The number of objects from the entire experiment recognized by the participant.
			Object association	The participants’ ability to group together objects that had been presented together in an episode.

Each stimulus presentation period started with a green screen (0.6 seconds), followed by the presentation of a unique random sequence of five objects presented for 0.6 seconds each, providing sufficient time for the participant to identify each object (Besson et al., 2012), before the episode ended with a red screen (0.6 seconds) (Fig. 1b). The temporal intervals between the objects and between the objects and the starting point (green screen) and end point (red screen) were randomly selected with a range between 0.1 and 2 seconds for three of the intervals and a range between 2.1 and 3.5 seconds for the remaining three intervals. This placed the objects in a unique temporal pattern, and ensured that the length of each episode was within the range of recommended block length for fMRI (Wager & Nichols, 2003).

The stimulus period was followed by a 10-second period when a fixation cross was present, during which the participants engaged in nonstimulus driven encoding (Clewett et al., 2019;N. Cohen et al., 2015) as confirmed by a questionnaire (“I continued memorizing the object sequence during cross fixation”: score 6.7 ± 2.0 (0: strongly disagree; 9: strongly agree)). The cross-fixation period was followed by an odd–even judgment task (10 seconds). During the odd–even task, the participants were instructed to push the right joystick button when an even number (<100) appeared on the screen and the left joystick button when an odd number (<100) appeared (numbers were presented at random). The participants were explicitly instructed to focus on getting the odd–even judgments correct. The purpose of the odd–even task was to prevent conscious processing of the episode (Wamsley, 2022). The relative short length of these consolidation periods was based on rest periods lasting only a few seconds benefiting consolidation (Ben-Yakov et al., 2013;N. Cohen et al., 2015;Wamsley, 2022).

Next, the participants were tasked with placing the objects from each episode at exact time points along an empty timeline demarcated with episode start and stop (*Timeline test_1_*) (Fig. 1c). This made it possible to measure the accuracy of precise timing and order in memory, as well as functional distortions such as temporal compression and expansion (Fig. 2;Table 1). Timeline test_1_was followed by another odd–even period. The purpose of this odd–even period was to provide an explicit break between the episodes, and to provide an implicit baseline for the fMRI data analysis.

After encoding and retrieval of a total of 16 episodes (one run), the participants were first tasked with remembering which objects had been presented together in an episode (*Object association test*) (Supplementary Fig. S1a). For each of the 16 episodes within a run, four of the five objects belonging to the episode were grouped together, and the participant was instructed to select the 5th object among 16 previously presented objects. The participant’s responses were scored as correct or incorrect, according to whether an object was correctly placed in the correct episode. Next, the participants were tasked with placing all episodes from the run in correct order on a timeline (*Timeline test_2_*) (Fig. 1d). In Timeline test_2_, the participant was shown an empty timeline representing the run just encountered. The participant was instructed to drag and drop each of the 16 episodes onto the timeline as accurately as possible based on when the episode was presented (Fig. 1d).

After having completed three runs, the participants were given an Object recognition test, a Run test, and a questionnaire related to strategies used to encode the episodes. In the Object recognition test, the participants were shown 385 pictures of various objects and were asked to identify the objects that had been presented during the experiment (a total of 240 objects) (*Object recognition test*) (Supplementary Fig. S1b). The participants were instructed to identify the objects they had encountered during the experiment by clicking on it (Supplementary Fig. S1b). The participants were given one point for each object correctly selected. In the Run test, the participant was shown an empty timeline representing the experiment. The participant was instructed to drag and drop the three runs onto the timelines as accurately as possible based on when in time the runs started. Finally, participants filled in a questionnaire with 22 questions about the strategies they used to encode and retrieve the object and episode sequences, to gauge whether the representations of time were associated with explicit encoding strategies or rather automatic processes beyond conscious control. The questionnaire had a 9-point scale, ranging from “strongly agree” (9) to “strongly disagree” (1). The participants indicated to what extent they agreed with the following statements: (1) I used rhythm to remember the object sequences; (2) I counted the number of seconds between the objects to remember the duration between the objects; (3) I used memory aids to remember the object sequences; (4) I gave the objects spatial positions; (5) I made up stories to connect the objects; (6) I thought about which categories the objects belonged to; (7) I thought about the names of the objects; (8) I thought about associations I had with the objects; (9) I imagined moving through an environment with the different objects at specific locations; (10) I created subgroups of objects within episodes; (11) I gave each object a number; (12) I continued memorizing the object sequence during cross-fixation; (13) I replayed the object sequence in my mind during cross-fixation; (14) I repeated the names of the objects during cross-fixation; (15) I compared the most recent object sequence with other object sequences during cross-fixation; (16) I started to think about the stimulus period during cross-fixation; (17) I started to think about something else during cross-fixation; (18) I relaxed during cross-fixation; (19) I continued memorizing the object sequence during the odd–even task; (20) I focused on the numbers during the odd–even task; (21) I started to think about something else during the odd–even task; and (22) I paid attention to object details.

BOLD fMRI was acquired from 36 of the participants continuously throughout the experiment, except for the periods when the participants performed the object association and timeline test across the episodes. The object recognition test, run-order test, and questionnaire were completed after the MRI scanning.

The web-based paradigm and tests were developed in Meteor (https://www.meteor.com/). The objects used in this paradigm were randomly selected from an archive, consisting of 480 high-quality normative color photographs of objects from a wide range of object categories (Brodeur et al., 2010;^2014^).

### 2.3 Precise timing measures within and between episodes

#### 2.3.1 Within episodes

Five within-episode precise timing measures were obtained from Timeline test_1_, namely (1) Translation_P1_, (2) Scaling_P1_, (3) Temporal pattern_P1_, (4) Object translation_P1_, and (5) Object scaling_P1_(Fig. 2;Table 1;Supplementary Fig. S2). (1) Translation_P1_is a measure of how accurately the participant positioned the objects in an episode relative to the boundaries of the episode. More precisely, Translation_P1_is a measure of the time difference between the geometric center of the recalled temporal pattern of all the objects and the geometric center of the correct temporal pattern. (2) Scaling_P1_indicates whether the participants compressed or expanded the time between all the objects. Scaling_P1_is, more precisely, a scaling factor that indicates whether the recalled time intervals between object presentations were globally compressed or expanded relative to the boundaries of the episode (or the intervals in the correct temporal pattern) (Fig. 2b). (3) Temporal pattern_P1_is a measure of how accurately the participants remembered the relative time between all the objects. This measure allows for both global scaling and translation of the object’s temporal pattern relative to the boundaries of the episode, that is, the green start and red end-of-episode screen color, and as such only considers the relative timing of object presentations (seeFig. 2b).

In more detail, each participant’s responses in Timeline test_1_were first translated such that the geometric center (centroid) of all the objects he/she had positioned on the timeline for an episode matched the center of the original (correct) temporal presentation of the objects. This was done by minimizing the root mean square deviation between the correct temporal pattern of the objects and the participant’s temporal pattern from the Timeline test (Kabsch, 1976) (seehttps://zpl.fi/aligning-point-patterns-with-kabsch-umeyama-algorithm/for a graphic illustration). Translation_P1_was defined as the inverse of this translation, and, therefore, reflects the participant’s ability to remember the temporal distance between the geometric center of the objects temporal pattern and the boundaries of the episode (green and red screens) (Fig. 2b;Table 1).

Next, the participant’s temporal pattern of the objects from the episode was scaled (up or down) to further minimize the root mean square deviation between the correct temporal pattern and the participant’s temporal pattern (Umeyama, 1991), see also (Evensmoen et al., 2021;Thorp et al., 2024). This second transformation, scaling, accounted for the fact that humans tend to either compress or expand temporal information in memory (Clewett et al., 2019;Eagleman, 2008;Howard, 2018). Based on this second transformation, we extracted Scaling_P1_, a scaling factor that reflects the extent to which the participant either compressed or expanded the timing between all the objects compared with the object’s original presentation.

We now had a version of the participant’s temporal pattern that had been translated and scaled relative to the correct temporal pattern of the objects. We then extracted the squared error between each position in the translated and scaled version of the participant’s temporal pattern and the closest position in the correct temporal pattern. These squared errors were then summed and multiplied by minus one to obtain the inverse of the total sum of squares error or Temporal pattern_P1_. Temporal pattern_P1_is, therefore, a measure of the overall ability of a participant to accurately recreate the temporal presentation or temporal pattern of all the objects within an episode, taking translation and scaling deviations into account.

At the level of individual object positions, Object translation_P1_was defined as the absolute deviation between each position in the temporal pattern of the objects from memory and the corresponding position in the correct temporal pattern. Object scaling_P1_is a scaling factor that evaluates whether the participant compressed or expanded the smallest time interval between each position in the temporal pattern from memory and the temporal position immediately preceding and/or following it, compared with the correct presentation of these objects (Fig. 2b).

#### 2.3.2 Between episodes

The test of memory of time between episodes was obtained from the Timeline test_2_using the same principles and methods employed for objects in an episode (Fig. 2c;Table 1). Five between-episode precise timing measures were obtained from Timeline test_2_, namely (1) Translation_P2_, (2) Scaling_P2_, (3) Temporal pattern_P2_, (4) Episode translation_P2_, and (5) Episode scaling_P2_. (1) Translation_P2_assessed the participants’ memory of when the temporal pattern of the episodes within a run started/ended relative to the correct start and end of a run. (2) Scaling_P2_evaluated whether the participants compressed or expanded the temporal intervals between the episodes in a run. Translation_P2_and Scaling_P2_were used to calculate (3) Temporal pattern_P2,_which estimated the degree of deviation between the episodes correct temporal pattern and the temporal pattern reconstructed by the participants from memory. Because the number of episodes within a run was larger (16) than the number of objects within an episode (5), we also estimated Temporal pattern_P2_, Translation_P2_, and Scaling_P2_for four consecutive episodes in a run (1-4, 5-8, 9-12, and 13-16). We also estimated Temporal pattern_P2_, Translation_P2_, and Scaling_P2_for an increasing number of consecutive episodes starting with the first episodes to episode 5 and onward (i.e., 1-5, 1-6, 1-7, 1-8, 1-9, 1-10, 1-11, 1-12, 1-13, 1-14, 1-15, and 1-16) within each run (Supplementary Fig. S4).

At the level of individual episodes, we first defined Episode translation_P2_, a measure that estimated the deviation between each position in the episodes’ temporal pattern in memory and the corresponding position in the episodes’ true temporal pattern. Second, we defined a scaling factor Episode scaling_P2_that evaluated whether the participants compressed or expanded the smallest interval between each position in the episodes’ temporal pattern in memory and the temporal position immediately preceding and/or following, compared with the true temporal pattern.

### 2.4 Order measures

Object order_O1_is a measure of how accurately the participant remembered the order or sequence positions of the objects in an episode. More precisely, it is a measure of the distance between the sequence position selected by the participant for each object in Timeline test_1_and the correct sequence position (Fig. 2b). Memory of the order between episodes, Episode order_O2_, reflects how accurately the participant remembered the order or sequence position of the different episodes in a run. It was estimated by calculating for each episode the deviation between the episode sequence positions as indicated by the participant and the correct sequence position in Timeline test_2_(Fig. 2c). If the participants, for example, indicated that the third episode within a run was the first episode, that episode would be given an Episode order score of 2 instead of a maximum deviation score of 0.

### 2.5 Memory for temporal and nontemporal aspects within and between episodes

To evaluate memory for temporal and nontemporal aspects of the episodes (Temporal pattern_P1_, Translation_P1_, Scaling_P1_, Temporal pattern_P2_, Translation_P2_, Scaling_P2_, Object order_O1_, Episode order_O2_, Object recognition, Object association), we compared the distribution of the test scores when using the participants correctly labeled responses with a shuffled distribution, for the whole sample of participants including the fMRI subsample. Both the actual response distribution and the shuffled distribution were based on participant averages.

**Fig. 3.**
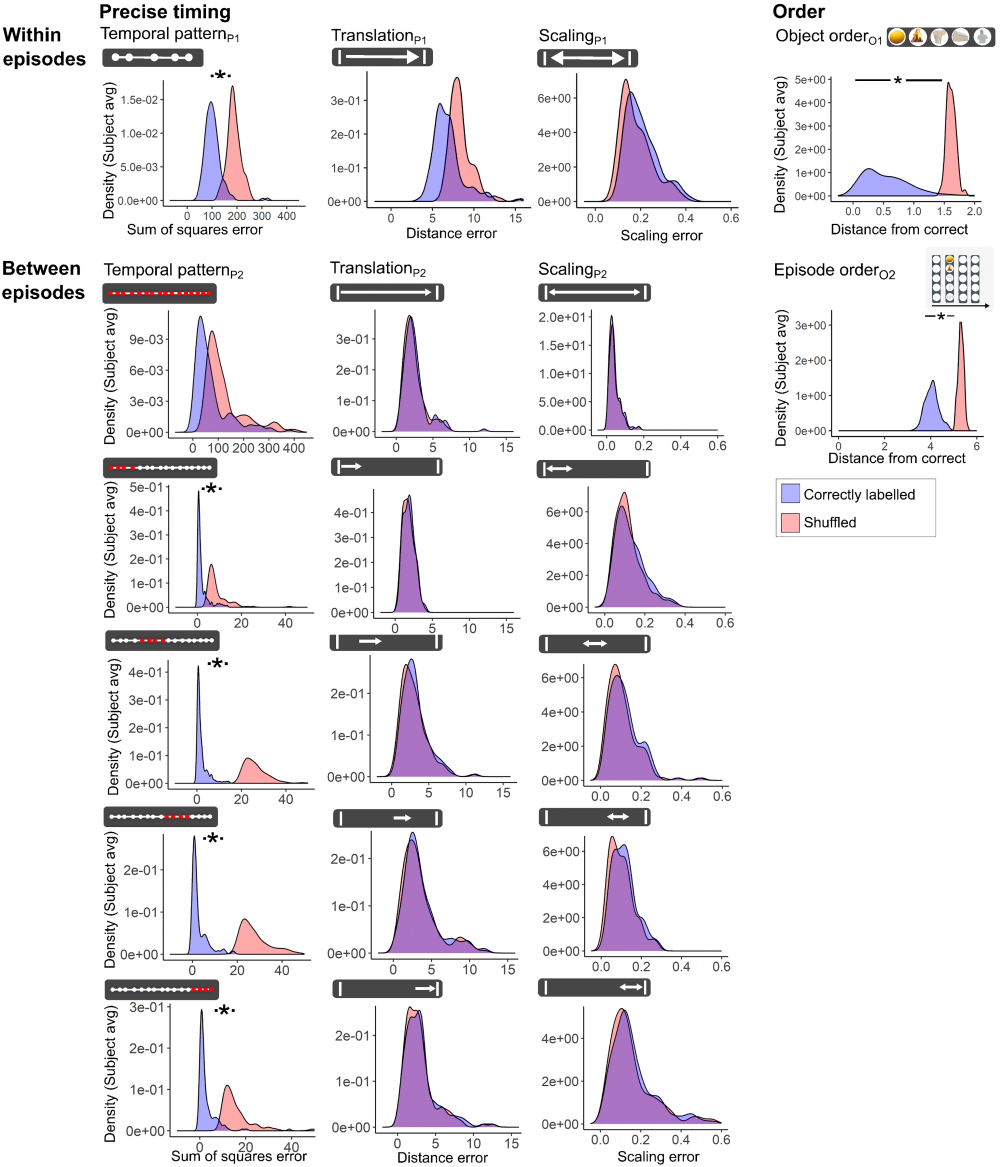
Precise timing is only represented on short timescales. The distribution of scores from Timeline test_1_and Timeline test_2_, based on “correctly labeled” responses (blue) compared with the “shuffled” distribution (red) (see alsoSupplementary Table S1; Methods). The distributions were based on the average score from each participant. Precise timing reflects the degree to which the participant’s response displays the accurate relative temporal pattern between objects within episodes (Temporal patternP1), or between all episodes as well as between four consecutive episodes (1-4, 5-8, 9-12, and 13-16) (Temporal patternP2) and the accurate translation and scaling between the objects or episodes and the temporal boundaries (see Methods;Fig. 2;Table 1). Between episodes, analyses using five consecutive episodes (1-5, 6-10, and 12-16) instead of four gave similar results. The shuffled distribution was estimated either by comparing the temporal pattern from memory with the correct temporal pattern from all the other episodes (measures of precise timing) (left) or by comparing random sequences of numbers with ordered sequences of numbers (measures of order) (right) (see alsoSupplementary Figs. S3 and S4;Supplementary Table S1; Methods). *p < 0.05 (FDR corrected).

For the precise timing measures within episodes, the correctly labeled distribution was estimated by comparing the temporal pattern produced by the participants for each episode with the correct temporal pattern. The shuffled distribution was estimated by comparing the temporal pattern produced by the participants for each episode with the correct temporal pattern from all the other episodes (ignoring the correct temporal pattern from the target episode) (Fig. 3;Supplementary Fig. S3). For the precise timing measures across episodes, the distributions were estimated in a similar way as within episodes. For the order measures, the correctly labeled distribution was estimated by comparing the object order from memory with the correct object order, and the shuffled distribution by comparing random sequences of numbers with ordered sequences of numbers 5000 times. For object recognition, we first identified all the objects selected by each participant in the Object recognition test including lures. Then five objects were sampled and the number of nonlures was counted. This procedure was repeated 50 times for each participant, and then repeated across all participants to establish the correctly labeled distribution (for the number of objects correctly recognized on average across episodes). The shuffled distribution was established by sampling five objects from the complete set of objects and lures (originally encountered by each participant) and then counting the number of lures. This was then repeated 5000 times. For Object association, the correctly labeled distribution was estimated by comparing the object associations from memory to the correct object associations, and the shuffled distribution was generated by comparing random responses (generated using random sequences of 16 numbers) with the correct response (an ordered sequence of the same 16 numbers).

To evaluate whether the correctly labeled and shuffled distributions were significantly different, we used R 4.3.1 (R Core Team, 2018) and the waddR package (https://github.com/goncalves-lab/waddR). The overlap between the two distributions was evaluated using the 2-Wasserstein distance, and 5000 bootstrapped samples combined with a generalized Pareto distribution approximation to get accurate p-values (Schefzik et al., 2021). The Wasserstein distance can be interpreted as the energy required to transform one distribution into another (Panaretos & Zemel, 2019). One of the main advantages with the Wasserstein distance is that it takes the whole distribution into account. This contrasts with many other distance measures that only evaluate the difference between individual points. Hence, the Wasserstein distance can detect small differences between random and real distributions. Further, the Wasserstein distance remains stable when the distributions are overlapping, and it is robust to noise (Bigot, 2020;Panaretos & Zemel, 2019;Panaretos & Zemel, 2020;Peyré & Cuturi, 2019). The significance threshold between the correctly labeled and shuffled distributions was corrected for the number of temporal and nontemporal measures tested using a 5% False Discovery Rate (FDR). We also compared the correctly labeled and shuffled distributions by using the cutpointr package in R (https://cran.r-project.org/web/packages/cutpointr/vignettes/cutpointr.html). We constructed a receiver operating characteristics (ROC) curve by plotting true positive fraction (sensitivity) versus true negative fraction (specificity) for different thresholds (or cutoff values) between the two distributions (Supplementary Fig. S3).

### 2.6 The relationship between timing, order, and nontemporal aspects of episodic memory

To evaluate whether there was any association between precise timing, order, and nontemporal aspects of episodic memory, we employed mixed linear models with maximum likelihood estimates on a trial-by-trial basis. The data were analyzed in R using the mixed linear model package lme4 (https://cran.r-project.org/web/packages/lme4/index.html) across the whole group of participants including the fMRI subsample. The R package sjPlot was used for visualization (https://cran.r-project.org/web/packages/sjPlot/index.html). In these analyses, the temporal and nontemporal measures (Temporal pattern_P1_, Translation_P1_, Scaling_P1_, Temporal pattern_P2_, Object order_O1_, Episode order_O2_, Object recognition, Object association) were used as response variables in separate models, while the explanatory variables were selected based on whether their inclusion improved the AIC (Akaike information criteria) value of the model by using the buildmer package (https://cran.r-project.org/web/packages/buildmer/index.html). In addition, we evaluated absolute measures of goodness-of-fit to determine whether the included variables were indeed informative (Mac Nally et al., 2018). We also estimated the variation inflation factors for each model in order to evaluate collinearity between the explanatory variables using the R package car (https://cran.r-project.org/web/packages/car/index.html). First, we tested for random intercepts across participants. The fixed effects explanatory variables tested for inclusion in the model were the temporal and nontemporal measures (Temporal pattern_P1_, Translation_P1_, Scaling_P1_, Temporal pattern_P2_(for episode 1-4, 5-8, 9-12, and 13-16 within each run), Translation_P2_, Scaling_P2_, Object order_O1_, Episode order_O2_, Object recognition, Object association) excluding the measure used as a response variable, the variability of the temporal intervals in the true temporal pattern within the episode, the variability of the temporal intervals in the true temporal pattern between episodes, the sequence number for each of the three runs, the sequence number for each of the episodes within each run (order), the duration from the first till the last object presented for each episode, the duration of each episode, time used on Timeline test_1_, scores on the self-reported strategies, age, sex, and finally, a binary variable indicating whether the participant took part in another fMRI study first (Evensmoen et al., 2015) and subsequently participated in this study. This analysis was first performed using linear relationships for Scaling_P1_or Scaling_P2_. Secondly, using nonlinear relationships for Scaling_P1_or Scaling_P2_. Lastly, by using Object translation_P1_, Object scaling_P1_, Episode translation_P2_and Episode scaling_P2_that evaluated translation and temporal compression and/or expansion at the level of individual objects and episodes. We tested nonlinear relationships for object number within episodes and episode number between runs to investigate primacy and recency effects, that is, whether memory was most accurate for the first and last part of an episode or run. The significance threshold was corrected for the total number of explanatory variables, across all models, using a 5% False Discovery Rate (FDR).

The mediation package in R (https://cran.r-project.org/web/packages/mediation/) (Tingley et al., 2014) and the mixed linear model package lme4 were used to test whether the association between a variable A and a variable B was casually mediated by another variable called the mediator. First, a mixed linear model (model 1) with variable A as the explanatory variable and variable B as a response variable was used to establish whether it was a relationship between variables A and B. Then, a second mixed linear model (model 2) with the potential mediator as the response variable and variable A as an explanatory variable was used to establish whether it was a relationship between the mediator and variable A. A third mixed linear model (model 3) with variable B as the response variable and variable A and the mediator as explanatory variables was then defined to test whether there was a significant relationship between variable B and the potential mediator while controlling for the effects of variable A (on variable B). The potential mediator had to explain more or other parts of the variance in variable A than in variable B. If variable A did not explain any of the variance in variable B in model 3, the mediation would be defined as complete. Finally, only if models 2 and 3 showed significant relationships, could the significance of the mediation effect be assessed. The mediation package calculated the mediation effect by combining models 2 and 3 and then evaluating the significance of the indirect effects between variables A and B via the mediator. Significance was evaluated by using a quasi-Bayesian Monte Carlo method with 5000 draws to estimate 95% confidence intervals.

Finally, Spearman’s rho was used to investigate the correlation between the temporal and nontemporal measures of memory (Temporal pattern_P1_, Translation_P1_, Scaling_P1_, Object translation_P1_, Object scaling_P1_, Temporal pattern_P2_(for episode 1-4, 5-8, 9-12, and 13-16 within each run), Translation_P2_, Scaling_P2_, Object order_O1_, Episode order_O2_, Object recognition, Object association, Episode translation_P2_, and Episode scaling_P2_) using only average values from each subject. This analysis was done in R using the package corrplot (Wei & Simko, 2024). The significance threshold was corrected for the total number of temporal and nontemporal measures included in the analysis using a 5% False Discovery Rate (FDR).

### 2.7 Image acquisition

Functional and anatomical MR images were acquired from a subgroup of 36 participants using a 32-channel Head Matrix Coil on a 3T Siemens Skyra scanner (Siemens AG, Erlangen, Germany). Foam pads were used to minimize head motion. The fMRI stimuli were presented using an LCD monitor with 1280 x 1024 resolution, and the participant using an MRI compatible joystick to make responses (Current Designs, Philadelphia, PA, USA). Before the experiment started, the participant was first allowed to familiarize himself with the presentation equipment and the joystick, and then completed practice episodes from the different experimental conditions. Scanning was commenced when complete task compliance was ensured.

T2*-weighted, blood-oxygen-level-dependent sensitive images were acquired during the temporal learning and Timeline test_1_, using a 2D echo-planar imaging pulse sequence with whole brain coverage. FOV = 220 mm x 220 mm, slice thickness = 3.0 mm (no gap), number of slices = 74, matrix = 74 x 74, yielding 3.0 x 3.0 x 3.0 mm^3^voxels (TR = 2570 ms, TE = 32 ms, flip angle = 90°). GRAPPA acceleration was used, with a factor of four. The lengths of the functional runs varied between 528 and 781 volumes, due to the variable length of the episodes and the self-paced nature of the test period. For anatomical reference, a T1-weighted (T1W) 3D volume was acquired using an MPRAGE sequence (TR = 2300 ms, TE = 2.94 ms, FOV = 256 mm x 256 mm x 192 mm, matrix 256 x 256 x 192 yielding a resolution of 1.0 x 1.0 x 1.0 mm^3^, flip angle = 8°).

#### 2.7.1 fMRI preprocessing

Preprocessing of the fMRI data was performed using fMRIPrep-22.1.0 (Esteban et al., 2019) (http://fmriprep.readthedocs.io/en/stable/index.html), a Nipype-based tool (http://nipype.readthedocs.io/en/latest/). Each T1-weighted volume was corrected for intensity nonuniformity using N4BiasFieldCorrection v2.1.0 and skull stripped using antsBrainExtraction.sh v2.1.0 (using the OASIS template) (ANTs v2.1.0,http://stnava.github.io/ANTs/). Brain surfaces were reconstructed using recon-all from FreeSurfer v6.0.0 (https://surfer.nmr.mgh.harvard.edu/fswiki), and the brain mask estimated previously was refined with a custom variation of the method to reconcile ANTs-derived and FreeSurfer-derived segmentations of the cortical gray matter of Mindboggle (https://mindboggle.info/). Spatial normalization to the ICBM 152 Nonlinear Asymmetrical template version 2009c (http://www.bic.mni.mcgill.ca/ServicesAtlases/ICBM152NLin2009) was performed through nonlinear registration with the*antsRegistration*tool of ANTs, using brain-extracted versions of both T1-weighted volume and template. Brain tissue segmentation of cerebrospinal fluid, white matter, and gray matter was performed on the brain-extracted T1-weighted image using fast (FSL v5.0.9). Functional data were motion corrected using*mcflirt*(FSL). This was followed by coregistration to the corresponding T1-weighted image using boundary-based registration with 9 degrees of freedom, using bbregister (FreeSurfer v6.0.0). Motion correcting transformations, BOLD-to-T1w transformation, and T1w-to-template (MNI) warp were concatenated and applied in a single step using*antsApplyTransforms*with Lanczos interpolation.

### 2.8 Activation pattern similarity analyses

The fMRI data were first subjected to a univariate (single-subject) analysis in SPM 12 (https://www.fil.ion.ucl.ac.uk/spm/software/spm12/). Brain activity was modeled using a general linear model (GLM) with the default options. Two models were used to investigate associations between scaling measurements (Scaling_P1_^Exp^, Episode scaling_P2_^Exp^, Scaling_P1_^Compr^, Episode scaling_P2_^Compr^) and activation pattern dissimilarity. Model one was used to investigate activation pattern dissimilarity between object-specific activation patterns within episodes and model two was used to investigate activation pattern dissimilarity between episodes. In model one, the explanatory variables were, in addition to intercept, one for each object event within the episodes for the stimulus period, as well as one for the cross-fixation period, one for the odd–even period that followed the cross-fixation period, one for the Timeline test_1_planning period, and one for the Timeline test_1_execution period, resulting in a total of 245 regressors. Each of the 240 object events was modeled using a hemodynamic response predictor which involved a canonical hemodynamic response function (two gamma functions). In model two, the explanatory variables were, in addition to intercept, one for each episode for the stimulus period, one for each episode for the cross-fixation period, one for each episode for the odd–even period that followed the cross-fixation period, one for each episode for the Timeline test_1_planning, and one for each episode for the Timeline test_1_execution period, resulting in a total of 241 regressors. The odd–even period after Timeline test_1_served as an implicit baseline for all three models. The effect of time points associated with abnormal shifts in signal change due to head motion or severe artifacts was removed from the analysis, by estimating the root mean square variance over voxels (DVARS) and including a regressor for each time point with an abnormal DVARS value (https://fsl.fmrib.ox.ac.uk/fsl/fslwiki/FSLMotionOutliers) (Caballero-Gaudes & Reynolds, 2017).

In order to test for associations between encoding accuracy and changes in activation patterns, we used a multivariate representational similarity analysis (Albouy et al., 2008;Nili et al., 2014), implemented in MATLAB (R2018a; MathWorks, Natick, MA, USA). The multivariate analysis was restricted to a gray matter mask described above to reduce noise and improve classification accuracy (Kriegeskorte et al., 2006;Oosterhof et al., 2011). The gray matter mask was based on the Harvard Oxford Structural Atlases and the MNI-fnirt cerebellar atlas (probability threshold set to 50%) (part of FSL;http://fsl.fmrib.ox.ac.uk/fsl/fslwiki/Atlases). A spherical “searchlight” was obtained for each voxel across the whole brain using a maximum radius of 10 mm to create searchlights consisting of 10 voxels (each 3 mm^3^). This resulted in an average searchlight radius of 4 mm, previously shown to be optimal for detection performance (Kriegeskorte et al., 2006). Nonspherical searchlights were allowed for voxels close to the borders of the gray matter mask to make sure that each searchlight contained the same number of voxels. The searchlights across the whole brain were investigated separately and independently of each other. A multivariate noise normalization was then applied to these activation maps, by first extracting the GLM residual for each voxel, then using those residuals to create a covariance matrix between all voxels, and finally using that covariance matrix to perform a spatial prewhitening of the regression coefficients (Walther et al., 2016). The next step was to generate pairwise activation pattern dissimilarity maps between objects within each episode (Fig. 7) and between all episodes (Fig. 8), using a Euclidean distance measure (equivalent to computing the Mahalanobis distance between activity patterns) (Walther et al., 2016).

Within episodes, to test whether the object-specific activation patterns from each episode became more similar when participants temporally compressed the objects temporal pattern within the episode compared with expanded, the activation pattern dissimilarity maps between object events from the same episode (from model one above) were correlated with a model predicting that the object-specific activation patterns within episodes would become more similar when the episode was compressed (Scaling_P1_^Compr^) compared with expanded (Scaling_P1_^Exp^) (Fig. 7).

Between episodes, to assess whether the activation pattern dissimilarity between episodes was modulated by temporal compression or expansion, the episode-wise activation pattern dissimilarity maps (from model two above) were correlated with a model representing the dissimilarity in temporal expansion between episodes (Episode scaling_P2_^Exp^, Scaling_P1_^Exp^) or the dissimilarity in temporal compression between episodes (Episode scaling_P2_^Compr^, Scaling_P1_^Compr^) (Fig. 8). Thus, for the central voxel of the searchlight region, a correlation score was obtained that expressed the degree to which the similarity of activation patterns centered around that voxel was consistently modulated by temporal expansion or compression.

Spearman’s rho was used to assess whether the observed activation pattern dissimilarities correlated with the level of temporal compression or expansion in memory for objects and episodes, because it also allows monotonic (not strictly linear) relationships (Nili et al., 2014). For the analysis within episodes, we also controlled for temporal proximity effects between objects by using a partial correlation method (Carlin et al., 2011). The partial correlation method involved defining the observed activation pattern dissimilarities between the objects as the dependent variable, while the independent variables included the level of temporal compression or expansion between the objects in memory and the difference in millisecond timing between the presentation of each object. Smoothing was performed on the single-subject level after estimation of the activation pattern dissimilarities, so as not to reduce the “spatial fine structure of the data” (Kriegeskorte et al., 2006), and the resulting correlation maps were smoothed with a Gaussian kernel of 6 mm. To test whether the correlation effects were significantly different from zero at a group level, the smoothed correlation maps were tested nonparametrically with one-sample t-tests using the program Randomise (Winkler et al., 2014), part of the FSL software package. Inference used cluster mass statistic (Bullmore et al., 1999), with a cluster forming threshold set at p = 0.001. Clusters were considered significant at p = 0.05, corrected for multiple testing using the nonparametric distribution of the maximum statistic.

## 3 Results

### 3.1 Precise timing is only encoded on short timescales

To evaluate whether precise timing and order were encoded within and between episodes for the whole group of 139 participants including the fMRI subsample, we compared the participants’ actual response distribution with a shuffled distribution using the 2-Wasserstein distance (Fig. 3; Methods). Both the actual response distribution and the shuffled distribution were based on participant averages. Within episodes, Temporal pattern_P1_(Table1) showed a significant segregation between the correctly labeled and shuffled distributions (Fig. 3;Supplementary Figs. S3 and S4;Supplementary Table S1). Between episodes, the effect for temporal pattern was highly significant across 4 consecutive episodes and weaker with an increasing number of consecutive episodes—up to 16 where the effect was no longer significant (seeSupplementary Fig. S4). For translation and scaling, the correctly labeled and shuffled distributions were not significantly different neither within nor between episodes, suggesting that accurate translation and scaling of the temporal patterns were not encoded by the participants. For order, a clear segregation between the correctly labeled distribution and the shuffled distribution was present both within episodes (Object order_O1_) and between episodes (Episode order_O2_) (Fig. 3;Supplementary Fig. S3b;Supplementary Table S1). Taken together, the results showed that precise timing was only encoded at a short timescale (below the maximum run duration of 41.1 minutes in this experiment), whereas order was encoded across all timescales in this experiment.

### 3.2 Temporal compression within episodes is associated with more accurate memory

To evaluate whether temporal*compression*and*expansion*of the objects presentation affect episodic memories for all participants including the fMRI subsample, we employed separate mixed linear models with a continuous measure of temporal compression–expansion within episodes (Scaling_P1_), temporal compression–expansion between episodes (Scaling_P2_^CE^), and each of the temporal encoding measures (Temporal pattern_P1_, Translation_P1_, Temporal pattern_P2_, Object order_O1_, or Episode order_O2_) as response variable (see Methods). The temporal scaling variables took a value between 0 and 1 for compression, 1 in the event of “no scaling”, and above 1 for expansion of the temporal pattern. The whole range of Scaling_P1_and Scaling_P2_data from compression to expansion was used.

**Fig. 4.**
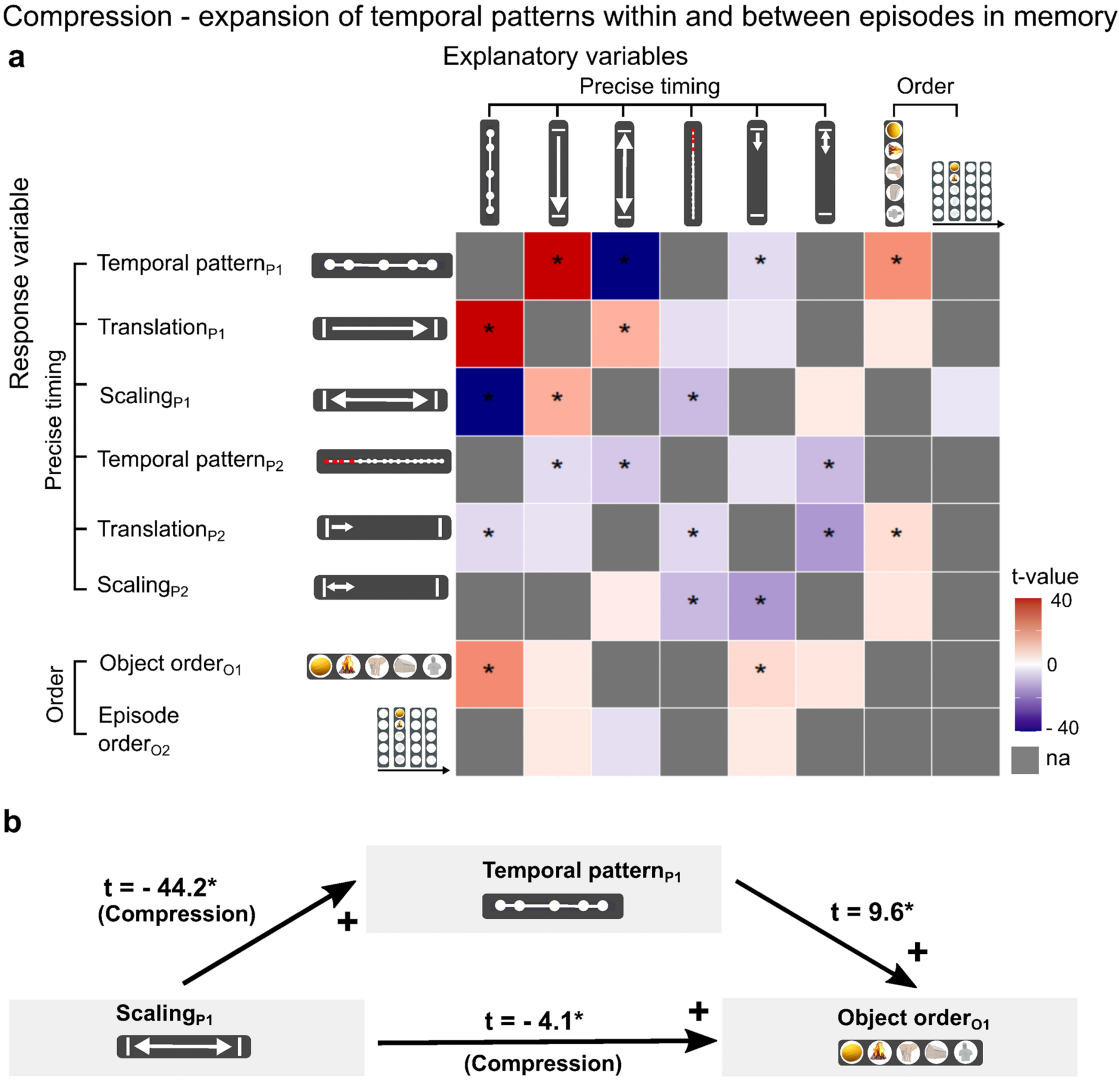
Temporal compression within episodes is associated with more accurate memory. The scaling variables used represented level of compression or expansion across all five objects within episodes and between four consecutive episodes. (a) Each row defines a behavioral mixed linear model with the row name defining the response variable and the column names defining the explanatory variables tested for inclusion in the model (see Methods;Supplementary Tables S3 and S4). (b) A causal mediation analysis was used to test whether more accurate memory for the Temporal pattern of objects within episodes (Temporal patternP1) mediated the effect of the scaling of the objects’ temporal pattern in memory (ScalingP1) on the accuracy of memory for object order (Object orderO1) (Average causal mediation effect: -0.14 (95% conf interval: -0.17, -0.11). *p < 0.05 (FDR corrected); “na” indicates the measure did not explain any additional variance.

The results showed that, within episodes, compression in memory of the temporal pattern (Scaling_P1_< 1) was associated with improved memory for Temporal pattern_P1_, and improved memory for Temporal pattern_P1_with improved memory for Object order_O1_(Fig. 4a;Supplementary Table S2). If the association observed between Scaling_P1_and Temporal pattern_P1_and between Scaling_P1_and Object order_O1_was simply related to deviation from correct, or zero scaling, we would expect to see a second-order nonlinear relationship for Scaling_P1_peaking when Scaling_P1_equals 1. However, there was no second-order nonlinear relationship with a peak when Scaling_P1_equals 1, confirming that the associations observed for Scaling_P1_were related to compression of the temporal pattern. When removing Temporal pattern_P1_from the mixed linear model analysis, compression of the encoded temporal pattern was associated with improved memory for Object order_O1_(Fig. 4b;Supplementary Table S3). These results indicate that the association between temporal compression of the episodes and more accurate object order memory is mediated by more accurate memory for the temporal pattern of the objects. A causal mediation analysis confirmed this (see Methods), as the positive effect of temporal compression within episodes on order accuracy was fully mediated by Temporal pattern_P1_(Fig. 4b). Between episodes, no association was observed between temporal compression or expansion and memory for order (Fig. 4a;Supplementary Table S2). Importantly, the estimated variation inflation factors for each model showed no evidence of a problem with multicollinearity among the explanatory variables (Supplementary Tables S2–S5), and Spearman’s rho showed low correlation between the participants’ average test scores (Supplementary Fig. S6). Finally, we found that memory for Object order_O1_was most accurate for the first part of the episode (primacy effect) and last part of the episode (recency effect) (Supplementary Fig. S5), consistent with memory being improved for the first and last words of a wordlist (Kljajevic et al., 2023;Murdock Jr, 1962). In contrast, memory for Episode order_O2_was highest for the mid part of the run, suggesting that primacy and recency effects only occur within episodes. The scores on the object recognition and object association tests both increased from run one to run three, suggesting a practice effect. Taken together, our main findings suggest that compression of time supports memory for objects within episodes (Fig. 6).

### 3.3 Temporal expansion between neighboring episodes is associated with accurate recall

**Fig. 5.**
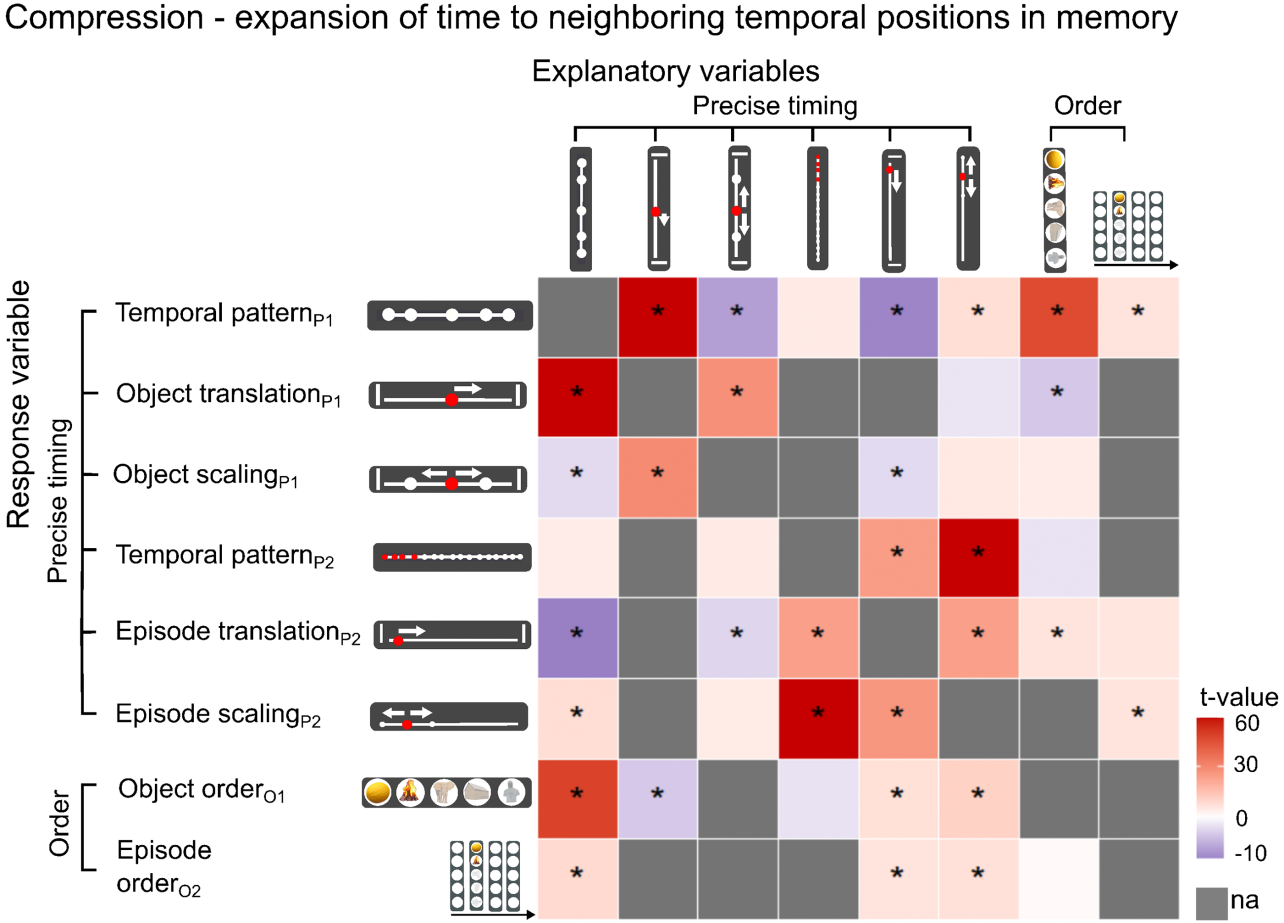
Temporal expansion to neighboring episodes is associated with more accurate memory. The scaling variables used evaluate level of compression or expansion of the time to the immediately preceding and/or following episode or object. The translation variables evaluate absolute deviation in the participant’s response compared with the correct position for each object or episode position. Each row defines a behavioral mixed linear model with the row name defining the response variable and the column names the explanatory variables tested for inclusion in the model (see Methods;Supplementary Table S4). *p < 0.05 (FDR corrected); “na” indicates that the measure did not explain individual variance in the response variable.

**Fig. 6.**
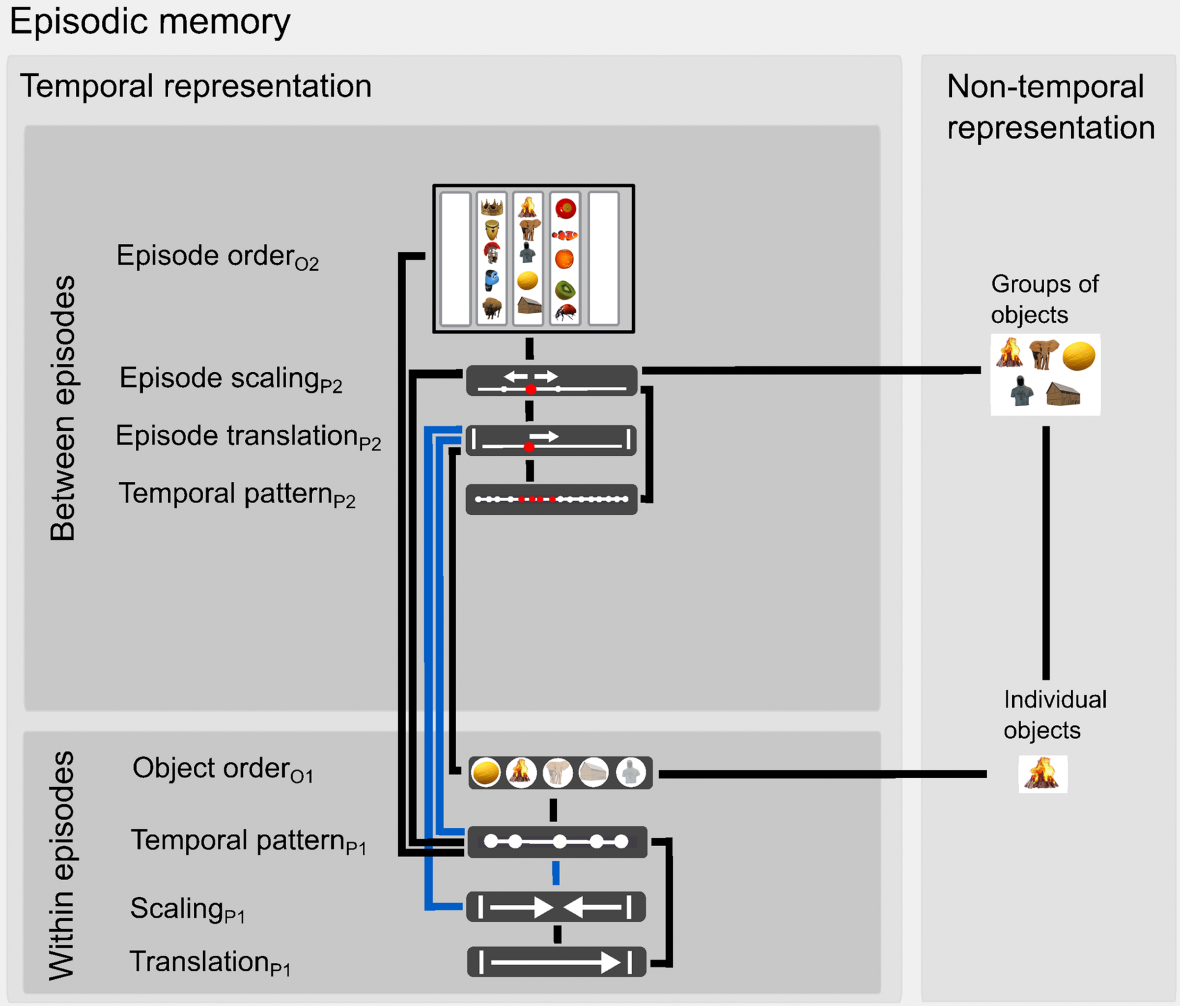
The relationship between the temporal and nontemporal features of episodic memories. The thick black lines indicate significant positive associations, and thick blue lines indicate a negative association, between the two measures in the mixed linear models (see alsoFigs. 4and5;Supplementary Figs. S5 and S6;Supplementary Tables S2–S4). All associations survived FDR correction for multiple comparisons at the 5% level.

Next, we investigated whether successful episodic memory is based on a more unique representation of individual object events or episodes, using temporal compression/expansion for singular objects and episodes as continuous variables (see Methods). At the level of individual objects within episodes, we found that compression between neighboring objects (negative Object scaling_P1_) was associated with accurate memory for the temporal pattern across all objects (Temporal pattern_P1_) and precise temporal position of the episode (Episode translation_P2_) (Fig. 5;Supplementary Table S4). While expansion of time between neighboring objects (positive Object scaling_P1_) was associated with a more accurate representation of the precise timing of the individual object (Object translation_P1_). This suggests that temporal compression to neighboring objects strengthens the memory for an episode as a whole, but also that the memory for an individual object within the episode is strengthened by temporal expansion to the neighboring objects. Between episodes, we found that expansion of time to neighboring episodes (Episode scaling_P2_) was associated with more accurate memory for timing within and between episodes (Temporal pattern_P1_, Temporal pattern_P2_), Episode order_O2_, and the precise temporal position of the episode (Episode translation_P2_), as well as Object association accuracy (Fig. 5;Supplementary Table S4). Summarized, these results suggest that the memory of an episode as a whole is improved by expansion of time to neighboring episodes and compression of time within the episode (Fig. 6).

### 3.4 fMRI activation pattern integration within episodes

Our next question was whether the compression of time within episodes was driven by activation pattern integration, in a whole brain fMRI dataset from a subgroup of 36 participants. We evaluated whether the observed behavioral link between temporal compression within episodes (Scaling_P1_) was driven by increased similarity between the object-specific activation patterns from the same episode using a multivoxel representational similarity analysis (RSA) (Nili et al., 2014;Walther et al., 2016).

**Fig. 7.**
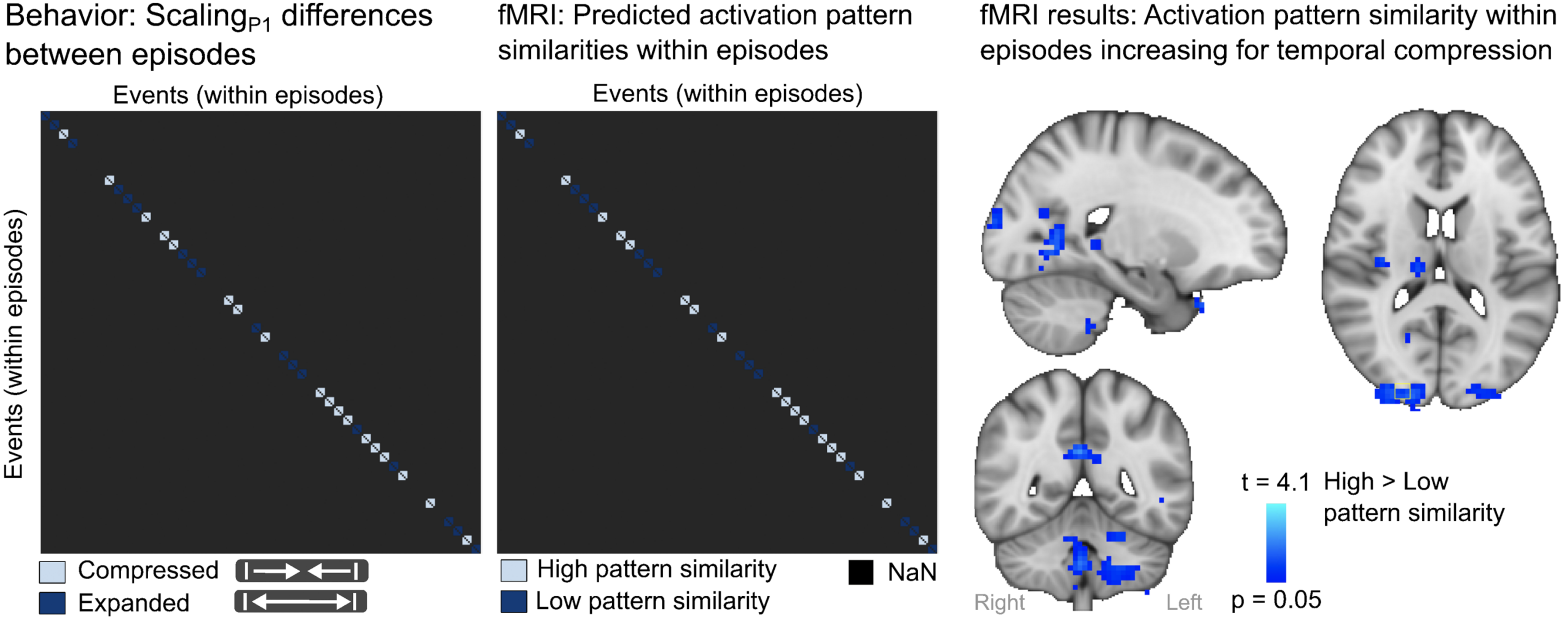
Activation pattern integration within episodes with temporal compression. Compression of the objects’ temporal pattern in memory compared with expansion (left) was predicted to be associated with increased similarity of object-specific activation patterns within episodes (mid). Voxels in the brain that showed more similar object-specific activation patterns within episodes with increased compression compared with expansion (right). Results are shown for the Stimulus period (seeFig. 1). For details on activation locations, seeSupplementary Table S5. p = 0.05 represents the cluster mass corrected thresholds.

For each 4-mm-radius searchlight across the whole brain, the RSA tested whether object-specific activation patterns were more similar when the objects’ temporal pattern within an episode was compressed (Scaling_P1_^Compr^) compared with expanded (Scaling_P1_^Exp^) and when the time to neighboring episodes was expanded (Episode scaling_P2_^Exp^) compared with compressed (Episode scaling_P2_^Compr^) (see Methods). Within episodes, the RSA revealed that temporal compression of the episodes compared with temporal expansion (Scaling_P1_^Compr^> Scaling_P1_^Exp^) was associated with more similar object-specific activation patterns within episodes in several brain regions including the posterior hippocampus (t = 3.7, p = 0.004), frontal pole (t = 3.9, p = 0.003), and orbitofrontal cortex (t = 3.9, p = 0.003) (Fig. 7;Supplementary Table S5). No effect was observed for Scaling_P1_^Exp^> Scaling_P1_^Compr^. Between episodes, no effect was observed (Episode scaling_P2_^Exp^> Episode scaling_P2_^Compr^, or Episode scaling_P2_^Compr^> Episode scaling_P2_^Exp^). Our fMRI results suggest that compression of the objects’ temporal pattern within episodes was driven by increasing activation pattern integration.

### 3.5 fMRI activation pattern differentiation between episodes

**Fig. 8.**
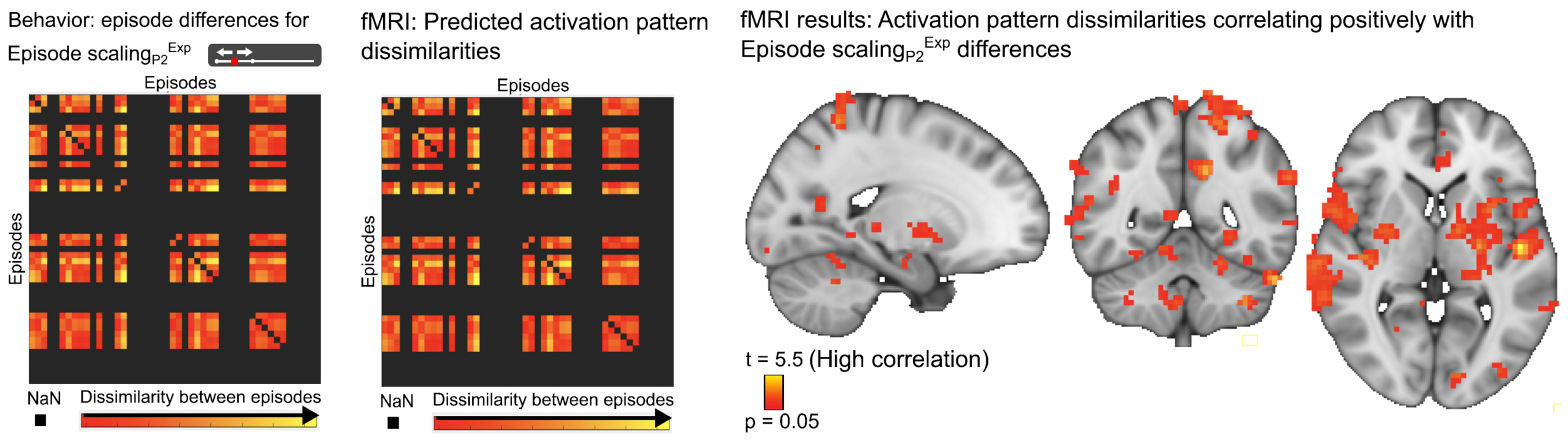
Activation pattern differentiation between episodes with expansion of time. The statistical model predicts that the activation pattern dissimilarity between episodes follows the dissimilarity in temporal expansion in memory to neighboring episodes (Episode scaling_P2_^Exp^) (left). Voxels in the brain that showed an activation pattern dissimilarity between episodes in the stimulus period that correlated with the dissimilarity in Episode scaling_P2_^Exp^(right). For details on activation locations, seeSupplementary Table S6. p = 0.05 represents the cluster mass corrected thresholds.

Finally, we asked whether the expansion of time between episodes was supported by activation pattern differentiation, by investigating activation patterns from searchlights across the whole brain. Our behavioral data showed that expansion of time between neighboring episodes was associated with more accurate memory for several temporal and nontemporal characteristics of the episode. This suggests that when an episode is given a distinct location on the timeline, by expanding the time to neighboring episodes, the memory for the episode becomes more accurate. Such expansion, or temporal pattern differentiation, could be driven by an increased activation pattern differentiation relative to other episodes. To test this, we investigated whether the dissimilarity in activation pattern from episode to episode reflected the episode-wise dissimilarity in temporal expansion (Episode scaling_P2_^Exp^, Scaling_P1_^Exp^) or temporal compression (Episode scaling_P2_^Compr^, Scaling_P1_^Compr^). Expansion of time to neighboring episodes (Episode scaling_P2_^Exp^) was related to the episode-wise dissimilarity in activation pattern in several brain regions including the anterior hippocampus (t = 3.9, p = 0.002), entorhinal cortex (t = 4.2, p = 0.02), middle frontal gyrus (t = 4.7, p = 0.001), inferior frontal gyrus (t = 4.3, p = 0.001), orbitofrontal cortex (t = 4.3, p = 0.03), and insula (t = 4.9, p = 0.001) (Fig. 8;Supplementary Table S6). No effect was observed for Scaling_P1_^Exp^, Episode scaling_P2_^Compr^, or Scaling_P1_^Compr^. These findings suggest that expansion of time between episodes is driven by increasing activation pattern differentiation.

## 4 Discussion

Within episodes, accurate memory for the order of the objects correlated positively with compression of time between the objects. Between episodes, accurate memory for episode order correlated positively with expansion of the time between neighboring episodes. Our fMRI data showed that compression of the duration of an episode was associated with more similar object-specific activation patterns within the episode. Expansion of time between neighboring episodes, however, was associated with more dissimilar activation patterns between episodes. Thus, behavioral as well as fMRI data support a dual model of episodic memory in which memory formation across objects within episodes is characterized by pattern integration, whereas memory formation between episodes is distinguished by pattern differentiation. These findings expand on existing models of episodic memory (Bellmund et al., 2020;Brown et al., 2007;Clewett et al., 2019;R. T. Cohen & Kahana, 2022;Cohn-Sheehy & Ranganath, 2017;Davachi & DuBrow, 2015;Farrell et al., 2011;Friedman, 2004;Howard & Eichenbaum, 2013;Howard & Kahana, 2002;Kurby & Zacks, 2008;Lee et al., 2020;Lohnas et al., 2015;Polyn et al., 2009;Talmi et al., 2019;Zacks, 2020), and lend strong support to the idea that episodic boundaries play a key role in the formation of successful episodic memories (Brunec et al., 2018;Clewett et al., 2019;Cohn-Sheehy & Ranganath, 2017;DuBrow & Davachi, 2013;Kurby & Zacks, 2008;Newtson & Engquist, 1976;Zacks et al., 2007).

### 4.1 The role of precise timing in episodic memory

We found that precise timing played a role in memory for order within episodes, driven by an association between the relative timing of the objects’ temporal pattern and object order. This is consistent with time-tagging theories, arguing that precise time labels are attached to objects during encoding (Friedman, 1993). Importantly, the relative timing measure used here allowed for scaling as well as translation of the temporal pattern relative to the boundaries of the episode, which means that the brain does not use absolute timing precision to encode an episodic memory. This observation is in line with the scalable temporal representations in the rodent hippocampus (Shimbo et al., 2021), and a constructed time beyond representation of order and precise timing in the human hippocampus (Bellmund et al., 2022). Furthermore, we found a positive association between the accuracy of the memory for the relative timing of objects’ temporal pattern and accurate memory for order within but not between episodes, suggesting that relative timing is more important when objects are close in time. If these assumptions are correct, representation of relative timing on a short timescale is a prerequisite for whatTsao et al. (2018)have called a “one shot formation of episodic memory.”

Between episodes, when comparing true and shuffled distributions, we found that the relative temporal pattern on average was only encoded for a period of less than 16 episodes or a duration of about 41 minutes. Thus, while relative timing may be crucial when successfully encoding the order of closely spaced objects, memory for episodes on larger timescales does not appear to depend on precise timing. Previously, short timescales have been defined as timing on the order of milliseconds to seconds and long timescales timing on the order of minutes (Jacobs et al., 2013;Montchal et al., 2019;Palombo & Verfaellie, 2017). However, these definitions of short and long timescales in memory are, as far as we know, not supported by studies that have actually measured the temporal duration when humans no longer recall precise timing. At larger timescales, it is not known whether the crucial parameter for memory is number of episodes or absolute time passed. It is possible that the estimate of the time passed between two episodes is based on the number of episodes in the intervening interval (Faber & Gennari, 2017;Jenkins & Ranganath, 2010;Lositsky et al., 2016). Our results suggest that counting the number of episodes to estimate duration becomes especially important for time periods that last more than 41 minutes.

### 4.2 Temporal compression and expansion

Compression of time between object presentations within an episode was associated with successful memory for the order in which the objects were presented. This is consistent with models suggesting that temporal context links items together (Howard & Kahana, 2002;Lohnas et al., 2015;Polyn et al., 2009), beyond direct item–item sequence links suggested by the associative chaining model (Lewandowsky & Murdock Jr, 1989). Previously, it has been observed that when humans re-experience episodic memories, the duration and timing of events in memory tend to be shorter than the objective (clock) time (Jeunehomme et al., 2018). The same is found when participants mentally navigate a familiar route through an environment (Bonasia et al., 2016;Brunec et al., 2017;Jafarpour & Spiers, 2017). In rodents, sequential replay of hippocampal place cell activity are likewise temporally compressed (Carr et al., 2011;Skaggs et al., 1996). Further, memory for order has often been observed to be enhanced within episodic boundaries (Pu et al., 2022). Our results suggest that compression of time within episodes is necessary for an enhanced representation of object order, and that this compression can be interpreted as a form of pattern integration of the objects’ temporal pattern.

It is well established that the interval between two items tends to be expanded across episodic boundaries in human memory (Brunec et al., 2018;Clewett et al., 2019,^2020^;Faber & Gennari, 2017). Here we show that expansion of time between neighboring episodes was associated with more accurate memory for several temporal and nontemporal characteristics of the episode, such as the objects’ temporal pattern, episode order, and object–episode associations. We suggest that our findings provide evidence of pattern differentiation on a larger timescale; episodes given a distinct location on the timeline are less susceptible to interference from other episodes and will be more accurately remembered. Our findings also support a proposed link between representation at short and long timescales (Lohnas et al., 2015).

### 4.3 fMRI activation pattern integration and differentiation

The present study demonstrates, using behavioral and neuroimaging data, that pattern integration and differentiation of individual episodes take place on different timescales during the formation of episodic memories. Hippocampal activation patterns for two objects in a sequence have been shown to become more similar with improved temporal order memory (DuBrow & Davachi, 2014). It is possible that this increase in hippocampal activation pattern similarity is driven by compression of the time between the objects rather than improved object order memory, as it has been shown that hippocampal activation pattern similarity also increases when two objects from the same sequence are perceived as being closer in time (Ezzyat & Davachi, 2014). Investigating temporal patterns within and between episodes instead of object pairs, our fMRI data showed that temporal compression of an episode was associated with increased activation pattern similarity for the objects within the episode, in several brain regions including the posterior hippocampus. In contrast, expansion of time between neighboring episodes was associated with increased pattern differentiation of episode-specific activation patterns in several brain regions including the anterior hippocampus. Previous investigations into spatial representation have suggested that the posterior hippocampus subserves fine-grained, local representations, whereas the anterior hippocampus subserves coarse, global representations (Evensmoen et al., 2013,^2015^;Poppenk et al., 2013;Xu et al., 2010). Our findings indicate that for time in episodic memory, the posterior hippocampus supports local representations and the anterior hippocampus supports more global representations, similar to what has been observed for spatial stimuli.

It has been proposed that the more detailed local representations in the posterior hippocampus are driven by pattern differentiation, while the coarse overview representations in the anterior hippocampus result from pattern integration (Poppenk et al., 2013). Our results instead provide evidence of pattern integration in the posterior hippocampus and pattern differentiation in the anterior hippocampus. Previous studies have observed memory-related activation pattern differentiation in both the posterior (Berens et al., 2018;Schlichting et al., 2015) and anterior hippocampus (Cowan et al., 2021;Ezzyat et al., 2018), and memory-related activation pattern integration in both the posterior (Cohn-Sheehy et al., 2021;Collin et al., 2015;Milivojevic et al., 2015) and anterior hippocampus (Collin et al., 2015;Ritchey et al., 2015). Anatomically, both the dentate gyrus associated with pattern differentiation and CA3 associated with pattern integration (Bein & Davachi, 2024;Treves & Rolls, 1994;Yassa & Stark, 2011) are located along the entire anterior-posterior hippocampal axis (Malykhin et al., 2010). Collectively, these results indicate that both pattern differentiation and pattern integration can take place along the entire anterior-posterior hippocampal axis.

The functional specialization with more local representations posteriorly and more global representations anteriorly has also been suggested for the cerebral cortex (Evensmoen et al., 2013;Kravitz et al., 2013). Concurring with this, we found that the largest cluster with increased activation pattern similarity with temporal compression within episodes was in the visual cortices, the most posterior part of the brain. In contrast, the largest cluster that showed increased activation pattern dissimilarity with increased temporal expansion between episodes involved more anterior brain regions such as the prefrontal cortex. This is consistent with activation pattern dissimilarity in the visual cortices being best predicted by a model consisting of many short movie events, and in more anterior brain regions by a model consisting of a few long events (Baldassano et al 2017).

We found that activation pattern dissimilarity in the entorhinal cortex was associated with expansion of time to neighboring episodes. Previous studies have found a role for the entorhinal cortex in representation of more accurate time and order in episodic or episodic-like memory in humans (Bellmund et al., 2019,^2022^;Montchal et al., 2019;Thavabalasingam et al., 2019;Umbach et al., 2020) and rodents (Heys & Dombeck, 2018;Kraus et al., 2015;Tsao et al., 2018). Our findings suggest that the entorhinal cortex is important for the construction of accurate episodic memories through functional temporal distortions.

The middle and inferior frontal gyrus and frontal pole showed more unique episode-specific activation patterns when time to neighboring episodes was expanded in memory. Previously, activation pattern dissimilarity between a target episode and neighboring episodes has been shown to be larger for accurate than for inaccurate trials in similar brain regions (Jenkins & Ranganath, 2010). Our data suggest that activation patterns in the prefrontal cortex not only support accurate episodic memories but also pattern differentiation of episodes through temporal expansion.

Activation patterns in the cerebellum were also connected to the compression of time within episodes. Cerebellum has previously been associated with reproduction of accurate temporal intervals (Teki & Griffiths, 2016). Further, the cerebellum is considered one of the core regions within the brain’s “main core timing network” (Merchant et al., 2013). Our results suggest that it is a closer link between the brain’s metric timing network and neural networks responsible for the representation of time in episodic memory than has previously been realized.

## 5 Conclusions

Successful memory for episodes is associated with distortions of time defined by compression of time within episodes and expansion of time between neighboring episodes. Moreover, object-specific brain activation patterns within episodes became more similar when the duration of an episode was compressed in memory. In contrast, brain activation dissimilarity between episodes was associated with temporal expansion between episodes in memory. In conclusion, the present behavioral and brain imaging findings support a dual process model of human episodic memory with pattern integration and differentiation taking place on different timescales for individual episodes. Further, our results suggest that the episodic memory system in our brain depends on clustering information through distortion of precise timing.

## Supplementary Material

Supplementary Material

## Data Availability

Access to data by qualified investigators is subject to scientific and ethical review and must comply with the European Union General Data Protection Regulations (GDPR), Norwegian laws and regulations, and NTNU regulations. Completion of a material transfer agreement (MTA) signed by an institutional official will be required to access the data. Access to the paradigm can be achieved through completion of a material transfer agreement (MTA) signed by an institutional official.
