# Supplementary material for: Pattern integration and differentiation: Dual process model of episodic memory
Source: Imaging Neurosci (Camb). 2025 Jan 9;3:imag_a_00433. doi: 10.1162/imag_a_00433 (PMC12319739; doi:10.1162/imag_a_00433)
Supplement: Supplementary Material [file imag_a_00433-supp.pdf]

Supplementary information

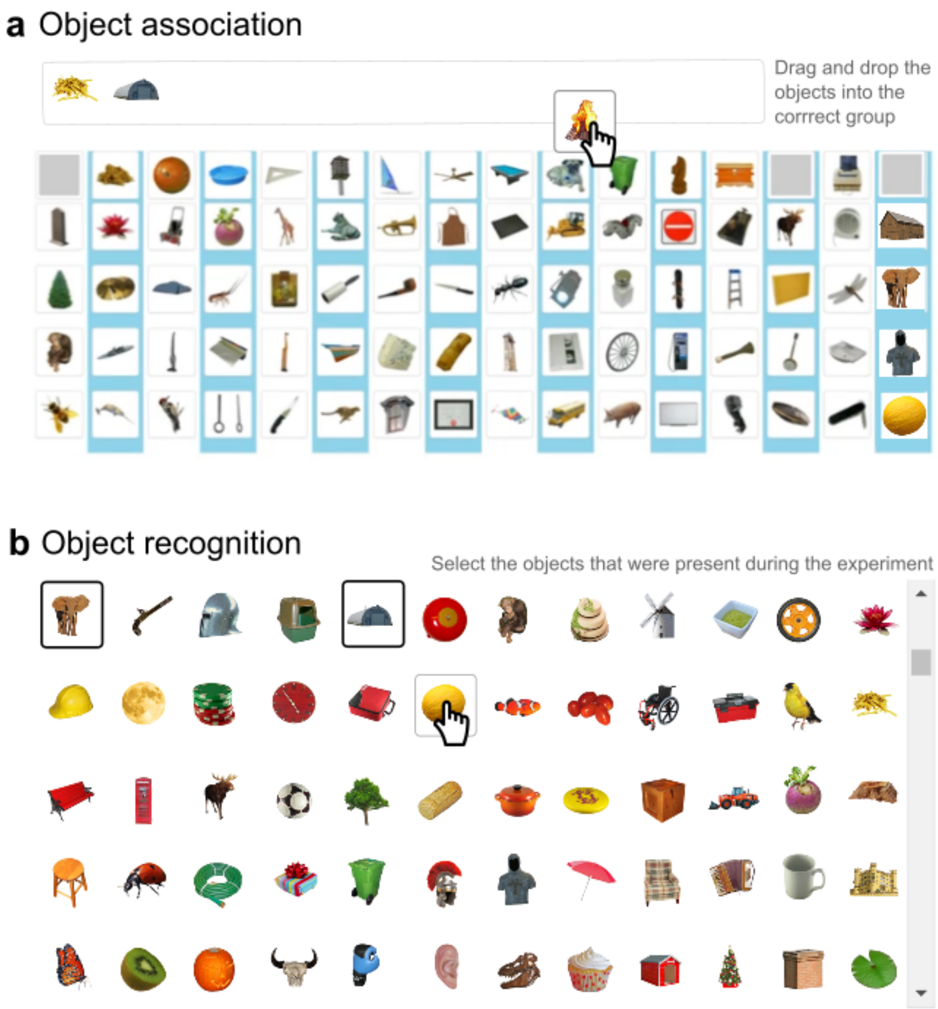

Figure S1. Non-temporal measures. **a**, After one run with presentation of sixteen episodes the participants were tasked with selecting which objects (or items) had been presented together (*Object association test*). **b**, Towards the end of the experiment after completion of all three runs, the participants were tasked with selecting the 248 objects that had been part of the experiment, among a total of 385 objects (*Object recognition test*).

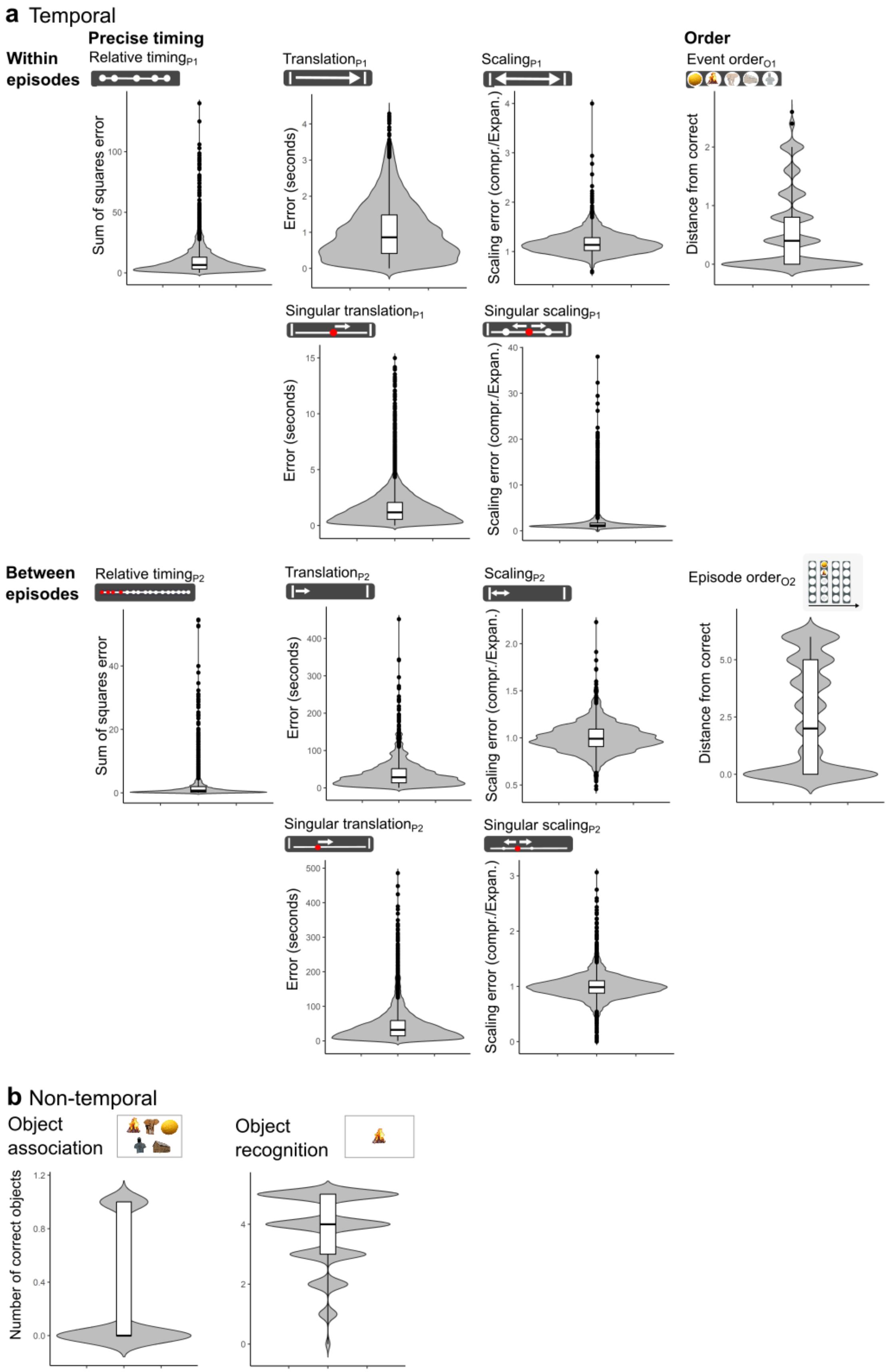

Figure S2. Memory for temporal and non-temporal aspects of episodic memories. **a**, Memory for the different temporal measures across all trials and all subjects within episodes (top rows) and between episodes (lower rows). Relative timing$_{P2}$, Translation$_{P2}$, and Scaling$_{P2}^{CE}$ are shown for four consecutive episodes (1-4, 5-8, 9-12, and 13-16. The results for scaling and compression reflect the level of compression or expansion of the temporal pattern in the participants responses and not absolute deviation from correct scaling (see Methods for a detailed explanation of the measures). **b**, Memory for the non-temporal measures.

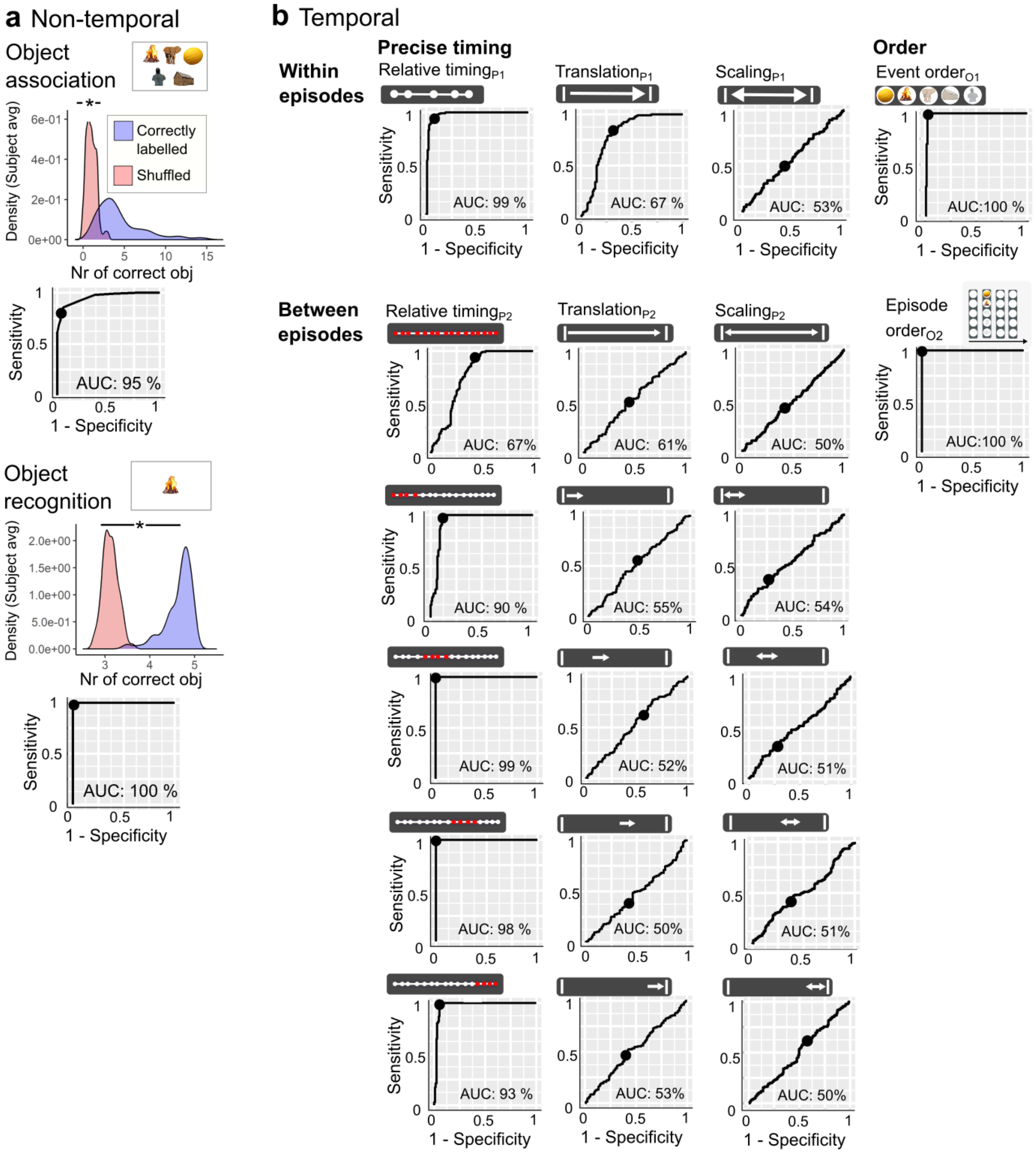

Figure S3. Correctly labelled vs shuffled distributions for the temporal and non-temporal aspects of episodic memory. The "correctly labelled" distribution of scores (blue) were compared to the "shuffled" distribution of scores (red). The distributions were based on the average score from each participant (see Figure 2 for density plots of the distributions for the temporal measures). Receiver operating characteristics (ROC) curve plots show true positive fraction (sensitivity) vs true negative fraction (specificity) for different cut-off values between the correctly labelled and shuffled distributions. Area under the curve (AUC) reflects the separability between the two distributions. An AUC value close to 1 (100%) indicates that the two distributions are highly separable, which means that the participants on average encoded the measure. The black circle reflects the cut-off value between the correctly labelled and the shuffled distribution that showed the most optimal sum of sensitivity (true positive fraction) and specificity (true negative fraction). **a**, Non-temporal measures including object recognition and object association (see Figure S1 and Methods). **b**, Temporal measures including relative timing, translation, scaling, and order (see Figure 2 and Table 1). Memory for non-temporal and temporal measures was assessed both within episodes (top row) and between episodes (lower rows). Analyses using five consecutive episodes (1-5, 6-10, and 12-16) gave similar results for precise timing accuracy between episodes.*$P < 0.05$ (FDR corrected).

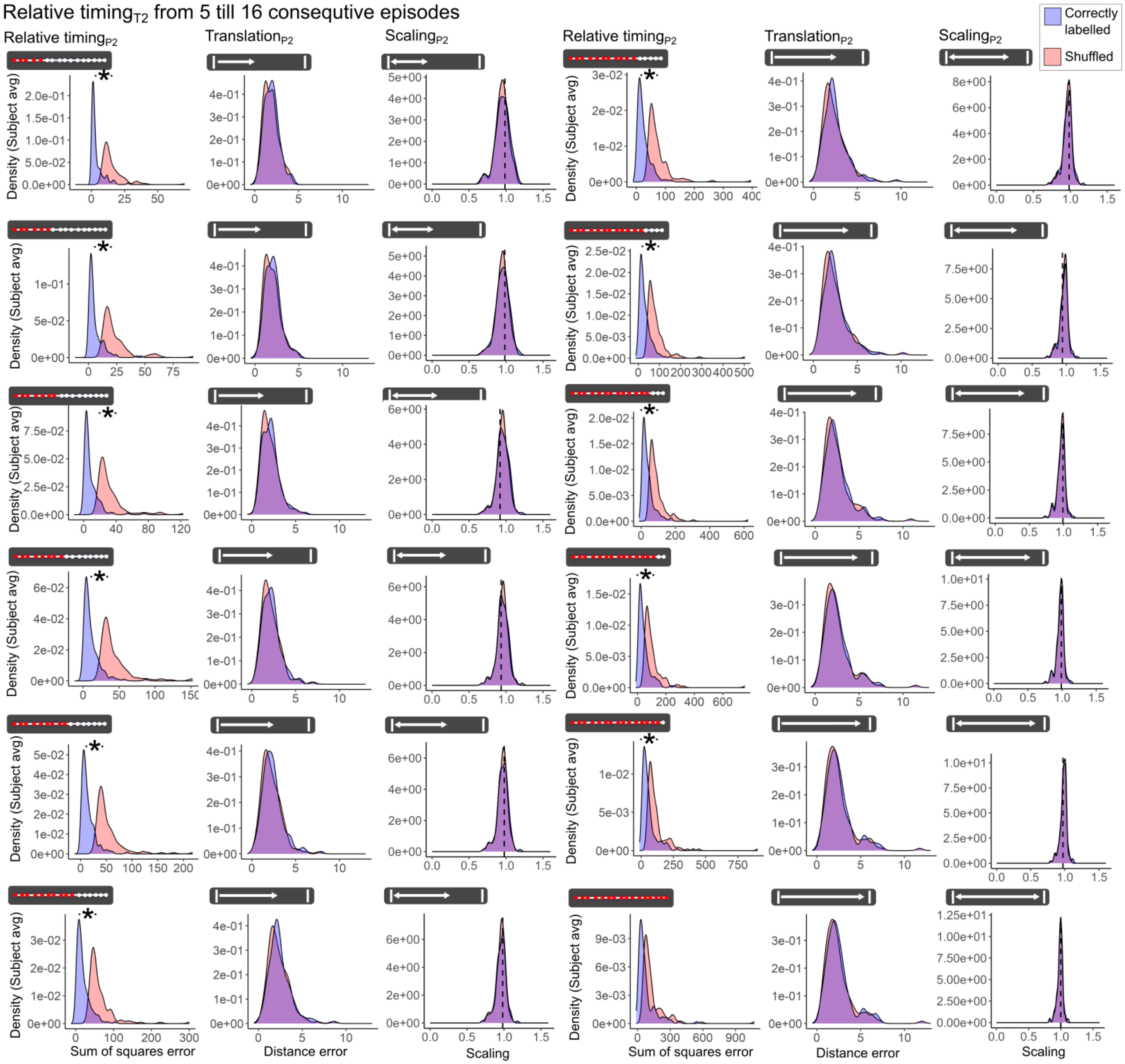

Figure S4. Representation of precise timing from 5 till 16 consecutive episodes. The "correctly labelled" distribution of scores (blue) compared to the "shuffled" distribution of scores (red) for 1-5 (top left panel), 1-6, 1-7, 1-8, 1-9, 1-10 (bottom left panel), 1-11 (from the top fourth panel), 1-12, 1-13, 1-14, 1-15, and 1-16 (from the bottom fourth panel) episodes within a run (see Methods). The distributions were based on the average score from each participant.*P < 0.05 (FDR corrected).

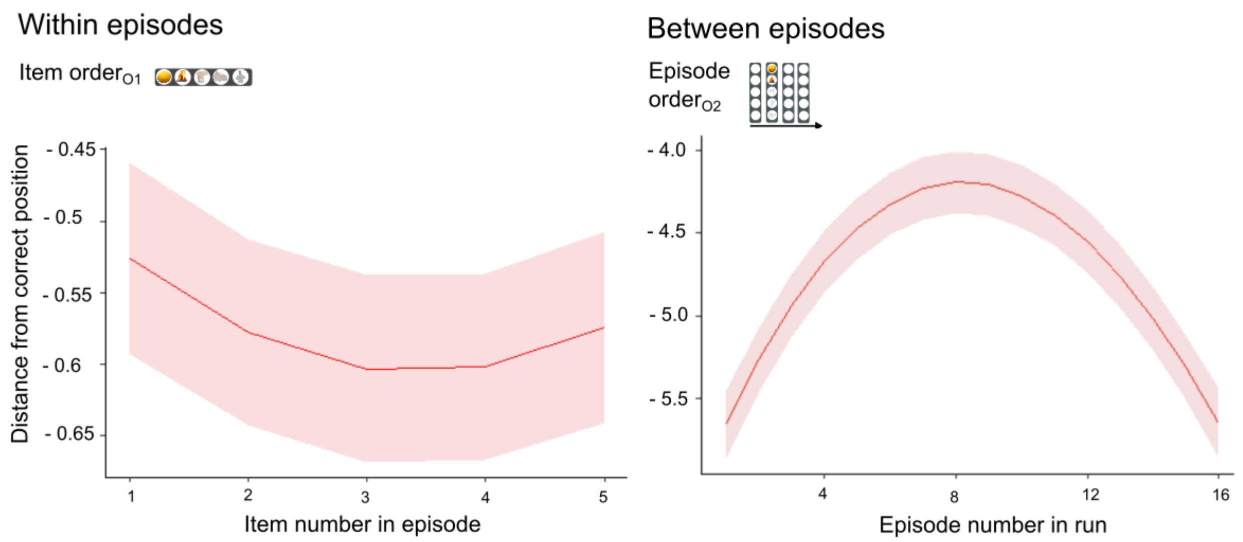

Figure S5. Primacy and recency effects. The model with Item order$_{O1}$ as a depent variable (Table S4) showed a positive second-order non-linear relationship with when during the episode that the item was presented (left) ($t = 5.1$, $p > 0.0001$). The model with Episode order$_{O2}$ as a dependent variable (Table S2) showed a negative second-order linear relationship with when during the run the episode was presented (right) ($t = -41$, $p > 0.0001$).

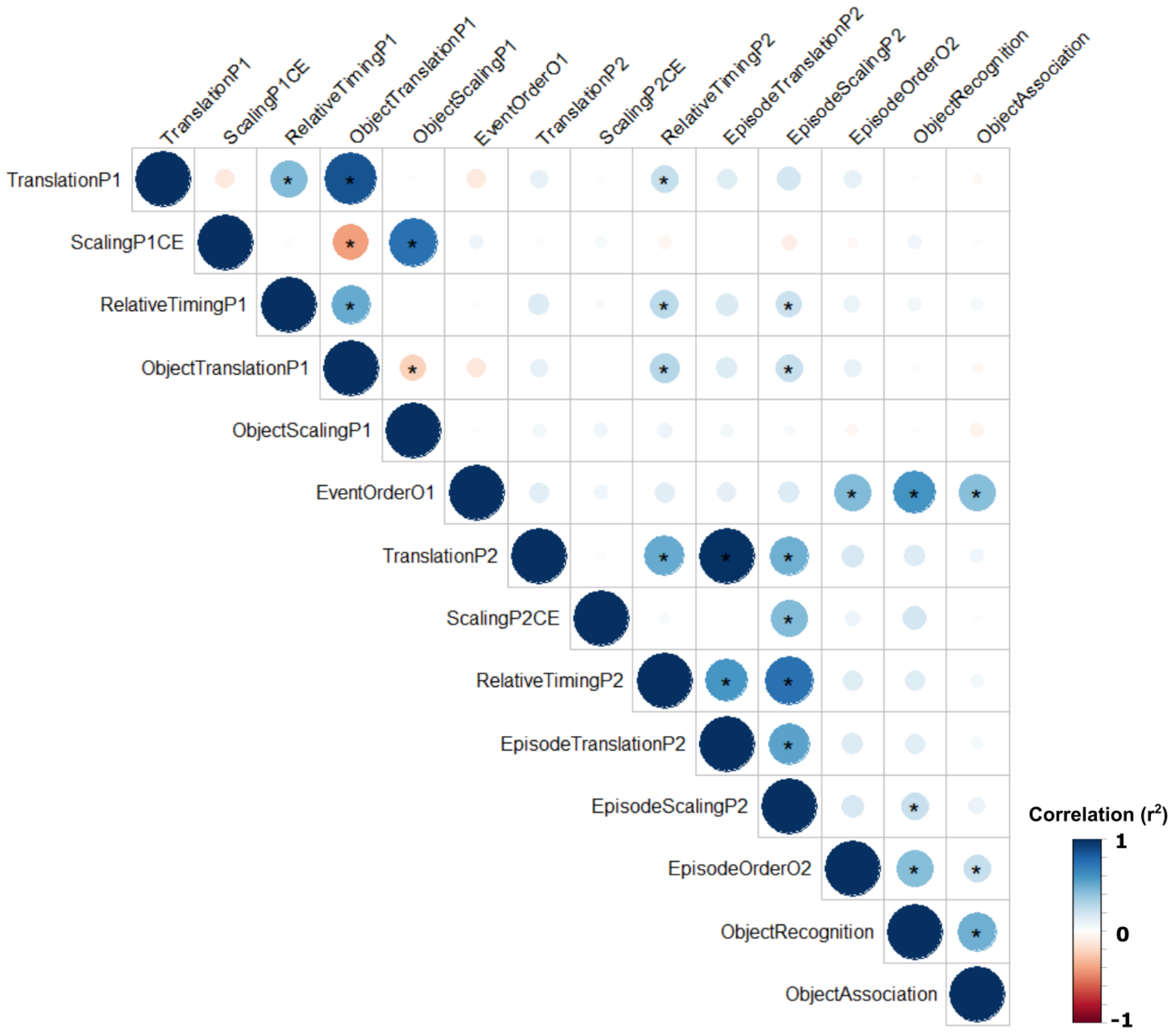

Figure S6. Correlation between measures of memory. Spearman's rho was used to estimate the correlation between the temporal and non-temporal measures across subjects, using the average memory measure score for each subject as input. The effect of Relative timing$_{P2}$ was evaluated using the accuracy across four consecutive episodes. The size of the correlation between each pair of memory measure is reflected both in the size and color intensity of each circle, with larger correlations being shown as darker and larger circles. *P < 0.05 (FDR corrected).

Table S1. Shuffled vs correctly labelled distribution for temporal and non-temporal measures.

	**2-Wasserstein distance²**	**p-value** (FDR corrected)	**Specific distribution differences** (%)		
			Location	Size	Shape
**Precise timing**					
Within episodes					
Relative timing$_{P1}$	7585.024	<0.0001*	99	0.16	0.45
Scaling$_{P1}$	0	0.9976	69	5.49	25.68
Translation$_{P1}$	2.557	0.215	92	4.97	2.57
Between episodes					
Relative timing$_{P2}$ (1 - 16)	5519.044	0.0557	64	25.57	10.71
Translation$_{P2}$ (1 - 16)	0.028	0.9972	31	6.08	62.75
Scaling$_{P2}$ (1 - 16)	0	0.9972	1	24	75.02
Relative timing$_{P2}$ (1 - 4)	51.465	<0.0001*	88	9.3	2.6
Translation$_{P2}$ (1 - 4)	0.008	0.9972	48	0.2	52.11
Scaling$_{P2}$ (1 - 4)	0	0.9972	36	37.88	25.88
Relative timing$_{P2}$ (5 - 8)	969.587	<0.0001*	81	18.7	0.37
Translation$_{P2}$ (5 - 8)	0.055	0.9972	67	2.2	30.74
Scaling$_{P2}$ (5 - 8)	0	0.9972	12	22.92	65.28
Relative timing$_{P2}$ (9 - 12)	1734.344	<0.0001*	52	44.04	3.6
Translation$_{P2}$ (9 - 12)	0.065	0.9972	43	0.54	56.76
Scaling$_{P2}$ (9 - 12)	0	0.9972	13	18.32	68.71
Relative timing$_{P2}$ (13 - 16)	2291.146	<0.0001*	13	82.64	4.49
Translation$_{P2}$ (13 - 16)	0.061	0.9972	41	11.23	47.42
Scaling$_{P2}$ (13 - 16)	0	0.9972	0	28.91	71.04
**Temporal order**					
Event order$_{O1}$	1.236	<0.0001*	93	7.39	0.1
Episode order$_{O2}$	1.621	<0.0001*	98	1.9	0.05
**Non-temporal measures**					
Object recognition	2.341	<0.0001*	99	0.95	0.52
Object association	17.033	<0.0001*	69	30.37	1.07

For each measure, overlap between the true and shuffled distribution was evaluated by using the 2-Wasserstein distance and 5000 bootstrapped samples combined with a generalized Pareto distribution approximation to get accurate p-values. In addition to the squared 2-Wasserstein distance, we also report how much the location, size, and shape of the distributions (in %) contributed to the observed squared 2-Wasserstein distance. *P < 0.05, corrected for multiple comparisons using a 5% False Discovery Rate (FDR).

Table S2. The relationship between temporal and non-temporal measures for $Scaling_{P1}^{CE}$ and $Scaling_{P2}^{CE}$

	Relative TimingP1		TranslationP1		ScalingP1CE		Relative TimingP2		TranslationP2		ScalingP2CE		Event orderO1		Episode orderO2		Object Recognition		Object association	
Predictors	*Est*	*stat*	*Est*	*stat*	*Est*	*stat*	*Est*	*stat*	*Est*	*stat*	*Est*	*stat*	*Est*	*stat*	*Est*	*stat*	*Est*	*stat*	*Est*	*stat*
(Intercept)	889.4 *	19.5	-12.7 *	-6.0	2.3 *	40.6	4.2 *	2.3	-1.8	-2.2	0.9 *	15.9	-1.0 *	-6.1	-4.8 *	-11.8	2.9 *	7.2	-0.2	-1.5
TranslationP1	12.4 *	42.3			0.0 *	6.7	-0.0 *	-2.3	-0.0	-1.9			0.0	1.6	0.0	1.8				
ScalingP1CE	-319.8 *	-44.2	2.0 *	6.6			-1.2 *	-3.7			0.0	1.6			-0.3	-2.1	-0.1	-2.2	-0.0 *	-2.3
Events duration	-6.7 *	-35.8	0.1 *	11.3	-0.0 *	-62.7					0.0 *	2.4	0.0 *	3.5					-0.0	-2.0
Q13 fix replayedSeq	6.2 *	2.3					0.2	2.2									0.1 *	2.9	0.0	1.7
TranslationP2	-1.6 *	-2.3	-0.0	-1.7			-0.1	-1.9			-0.0 *	-7.1	0.0 *	3.0	0.0	1.7			-0.0	-1.0
Temporal variabilityP1	-140.3 *	-20.3	1.7 *	6.5	-0.1 *	-14.2							0.1 *	2.7						
Event orderO1	30.7 *	9.6	0.2	1.8					0.2 *	2.7	0.0	2.1					0.2 *	8.7	0.0	1.6
Event test duration	0.0	0.3	0.0 *	2.3	-0.0	-0.5	-0.0 *	-4.5	-0.0	-0.5	-0.0 *	-2.9	-0.0	-1.5	0.0	0.6	0.0	0.0		
Q18 fix relaxed	-4.7	-1.5	0.2	1.9			0.2	1.9	-0.2 *	-2.6							-0.1	-2.1		
Q2 countedSec	-4.0	-1.4					0.3 *	3.2	0.1	1.6									0.0	2.1
Q7 objName	-3.7	-1.4			0.0	1.6	0.2	1.9					0.0	2.2			0.1 *	3.0	0.0	2.1
Q19 OE learnSeq	5.5	1.6																	-0.0	-1.7
Q20 OE focused	5.0	1.5			-0.0	-1.5	0.2	1.8			0.0	1.7			-0.1	-2.1			-0.0	-1.5
Episode nr in run	0.9 *	2.3			0.0	2.2							-0.0 *	-4.9			0.0	1.4		
Episode duration	-0.0 *	-2.9	0.0 *	2.4	0.0	1.5			-0.0	-1.8	0.0	1.9	-0.0 *	-2.3						
RelativeTimingP1			0.0 *	42.5	-0.0 *	-44.6			-0.0 *	-2.5			0.0 *	10.0						
Q12 fix seqLearn			0.5 *	3.9					0.1	1.8			-0.0	-1.6			-0.1 *	-2.6		
RelativeTimingP2			-0.0	-2.1	-0.0 *	-4.5			-0.0 *	-2.5	-0.0 *	-4.6								
Age			-0.2 *	-2.7															0.0 *	2.3
Q14 fix objNames			-0.2 *	-2.6			-0.2	-2.1	-0.1 *	-2.6			0.0 *	4.6						
Q6 objCat			-0.1	-1.5																
Q16 fix ThoughtStim			-0.1	-1.5									-0.0	-2.0						
Group					-0.1 *	-3.2	1.8 *	3.6	0.5	1.9	0.0	1.6			0.4 *	3.0				
Sex					-0.1 *	-2.9					0.0	2.2	0.1 *	2.3						
Q17 fix mindWander					-0.0 *	-2.5	-0.2	-1.8					-0.0	-1.6						
Q8 objAss					0.0	1.8	-0.2	-2.0							0.0	1.6				
Object association					-0.0	-2.1							0.0	1.4	0.2	1.7	0.2 *	7.2		
Episode orderO2					-0.0	-1.6														
ScalingP2CE					0.0	1.7	-2.2 *	-4.5	-1.1 *	-7.1			0.1	2.0			-0.1	-1.8		
Temporal variabilityP2							-12 *	-77	-0.2 *	-2.3	-0.1 *	-17.1	0.0 *	2.6			-0.0	-2.1		
Episode nr in run [1st deg.]							-41 *	-5.1	-34.7 *	-13.2	4.1 *	20.0			-1.3	-0.4			5.4 *	13.1
Episode nr in run [2nd deg.]							-15	-1.9	37.4 *	14.7	2.1 *	10.2			-40.5 *	-13.1			4.9 *	11.8
Q10 objSubGrp							-0.3 *	-2.8					-0.0	-2.1						
Run nr							-0.7 *	-6.0	-0.2 *	-6.2			-0.1 *	-12.6	-0.1	-1.6	0.2 *	12.7	0.0 *	4.9
Q9 imgMovEnv							0.2	1.9					0.1 *	6.0	0.1	2.0	0.1	2.2	0.0 *	2.3
ObjectRecognition											-0.0	-1.8	0.1 *	9.3					0.0 *	7.7
Q5 stories											-0.0	-1.8							0.0 *	5.0
Q4 spatialPos											0.0	1.4			0.0	1.8				
Q21 OE mindWander											0.0	1.5	0.0 *	3.2						
Q22 objDetails													-0.0	-2.0	0.1	2.1	0.1 *	2.5		
Q15 fix compSeq															-0.1	-2.0	-0.1	-1.6		
Q11 objANumber																			-0.0	-2.1
Q1 rythm																			0.0	1.5
**Random Effects**																				
σ^2	20740.91		29.13		0.05		57.72		6.19		0.04		0.29		8.84		0.82		0.16	
τ_{00}	5551.11 Id		6.44 Id		0.01 Id		5.86 Id		2.29 Id		0.01 Id		0.06 Id		0.35 Id		0.61 Id		0.01 Id	
ICC	0.21		0.18		0.18		0.09		0.27		0.14		0.17		0.04		0.43		0.08	
N	137 Id		137 Id		137 Id		137 Id		137 Id		137 Id		137 Id		137 Id		137 Id		137 Id	
Observations	6562		6562		6562		6562		6562		6562		6562		6562		6562		6564	
Marginal R^2 / Conditional R^2	0.529 / 0.629		0.357 / 0.473		0.488 / 0.578		0.582 / 0.621		0.101 / 0.344		0.160 / 0.277		0.211 / 0.346		0.042 / 0.078		0.170 / 0.524		0.119 / 0.193	
**VIF (avg(range))**	1.1(1-1.42)		1.2(1-1.84)		1.11(1-1.39)		1.14(1-2)		1.16(1-2.38)		1.19(1-2.3)		1.26(1-2.16)		1.08(1-2)		1.17(1.01-1.4)		1.11(1-2)	

In these analyses, the temporal and non-temporal measures were employed as response variables in separate models, and explanatory variables were selected on the basis of whether their inclusion improved the second-order AIC (Akaike information criteria) value of the model. In addition, we evaluated absolute measures of goodness-of-fit to determine whether the included variables were indeed informative. We also estimated the variation inflation factors for each model in order to evaluate collinearity between the explanatory variables. The effect of Relative $timing_{P2}$ was evaluated using the accuracy across four consecutive episodes. Two participants were excluded from these analyses as they did not fill out the questionnaire related to the strategies used to encode and retrieve the object and episode sequences. Further, a few observations were defined as clear outliers based on visual inspection and therefore removed from the analyses. VIF = variance inflation factor. $P < 0.05$, corrected for multiple comparisons using a 5% False Discovery Rate (FDR).

Table S3. The association between temporal and non-temporal measures for $Scaling_{P1}^{CE}$ and $Scaling_{P2}^{CE}$ without the Relative timing measure.

	TranslationP1		ScalingP1CE		TranslationP2		ScalingP2CE		Event orderO1		Episode orderO2		Object Recognition		Object association	
Predictors	*Est*	*stat*	*Est*	*stat*	*Est*	*stat*	*Est*	*stat*	*Est*	*stat*	*Est*	*stat*	*Est*	*stat*	*Est*	*stat*
(Intercept)	2.6	0.8	2.3*	17.6	-2.2*	-2.6	0.9*	16.0	-0.9*	-7.8	-4.8*	-11.8	2.9*	7.2	-0.2	-1.5
ScalingP1CE	-4.2*	-13.9					0.0	1.8	-0.1*	-4.1	-0.3	-2.1	-0.1	-2.2	-0.0*	-2.3
Q12 fix seqLearn	0.7*	3.7			0.1	1.7							-0.1*	-2.6		
Temporal variabilityP2	0.3	2.1	0.0*	3.0			-0.1*	-19.1	0.0*	2.6			-0.0	-2.1		
Events duration	-0.0*	-4.4	-0.0*	-49.8	0.0	1.4	0.0*	2.4							-0.0	-2.0
Event orderO1	0.8*	6.2	-0.0*	-3.7	0.1*	2.4	0.0	2.0					0.2*	8.7	0.0	1.6
Q14 fix objNames	-0.4*	-2.9			-0.1*	-2.6			0.0*	4.3						
Age	-0.2	-2.0	-0.0	-1.6											0.0*	2.3
Event test duration	0.0*	3.0	0.0	0.0	-0.0	-0.7	-0.0*	-2.6	-0.0	-1.5	0.0	0.6	0.0	0.0		
Temporal variabilityP1	-0.9*	-3.0	-0.1*	-5.4												
TranslationP2	-0.1*	-3.5					-0.0*	-7.0	0.0*	2.7	0.0	1.7			-0.0	-1.0
ScalingP2CE	0.6	1.5	0.0*	2.6	-1.1*	-6.9			0.1	2.0			-0.1	-1.8		
Run nr	-0.2	-1.9	0.0*	2.7	-0.2*	-6.0			-0.1*	-12.9	-0.1	-1.6	0.2*	12.7	0.0*	4.9
TranslationP1			-0.0*	-14.2	-0.0*	-3.4			0.0*	6.6	0.0	1.8				
Group			-0.1	-2.0	0.5	1.8	0.0	1.5			0.4*	3.0				
Sex			-0.1	-2.1			0.0	2.2	0.1*	2.4						
Q7 objName			0.0	1.7					0.0	2.0			0.1*	3.0	0.0	2.1
Q17 fix mindWander			-0.0	-2.0												
Q20 OE focused			-0.0	-1.9			0.0	1.7			-0.1	-2.1			-0.0	-1.5
Q11 objANumber			0.0	1.5											-0.0	-2.1
Q8 objAss			0.0	1.7							0.0	1.6				
Q13 fix replayedSeq			-0.0	-1.5									0.1*	2.9	0.0	1.7
Episode duration			0.0*	3.3	-0.0	-1.7	0.0	1.8	-0.0*	-2.7						
Object association			-0.0	-2.2					0.0	1.4	0.2	1.7	0.2*	7.2		
Episode orderO2			-0.0	-1.9												
Q18 fix relaxed					-0.2*	-2.6							-0.1	-2.1		
Episode nr in run [1st deg.]					-34.5*	-13.1	4.1*	20.3			-1.3	-0.4			5.4*	13.1
Episode nr in run [2nd deg.]					37.6*	14.7	2.1*	10.3			-40.5*	-13.1			4.9*	11.8
Q2 countedSec					0.1	1.5									0.0	2.1
ObjectRecognition							-0.0	-1.8	0.1*	9.5					0.0*	7.7
Q5 stories							-0.0	-1.8							0.0*	5.0
Q4 spatialPos							0.0	1.4			0.0	1.8				
Q21 OE mindWander							0.0	1.6	0.0*	2.9						
Q9 imgMovEnv									0.1*	6.0	0.1	2.0	0.1	2.2	0.0*	2.3
Q16 fix ThoughtStim									-0.0	-2.1						
Q22 objDetails									-0.0	-2.1	0.1	2.1	0.1*	2.5		
Q10 objSubGrp									-0.0	-1.6						
Episode nr in run									-0.0*	-4.7			0.0	1.4		
Q15 fix compSeq											-0.1	-2.0	-0.1	-1.6		
Q1 rythm															0.0	1.5
Q19 OE learnSeq															-0.0	-1.7
**Random Effects**																
σ^2	36.20		0.06		6.20		0.04		0.29		8.84		0.82		0.16	
τ_{00}	19.90 $_{Id}$		0.02 $_{Id}$		2.28 $_{Id}$		0.01 $_{Id}$		0.07 $_{Id}$		0.35 $_{Id}$		0.61 $_{Id}$		0.01 $_{Id}$	
ICC	0.35		0.27		0.27		0.13		0.19		0.04		0.43		0.08	
N	137 $_{Id}$		137 $_{Id}$		137 $_{Id}$		137 $_{Id}$		137 $_{Id}$		137 $_{Id}$		137 $_{Id}$		137 $_{Id}$	
Observations	6562		6562		6562		6562		6562		6562		6562		6564	
Marginal R^2 / Conditional R^2	0.086 / 0.410		0.284 / 0.479		0.099 / 0.341		0.156 / 0.270		0.189 / 0.340		0.042 / 0.078		0.170 / 0.524		0.119 / 0.193	
**VIF (avg(range))**	1.16(1.01-1.73)		1.07(1-1.25)		1.11(1.01-1.28)		1.22(1-1.85)		1.18(1-2.1)		1.16(1.01-1.37)		1.16(1.01-1.44)		1.3(1.05-2.02)	

In these analyses, the temporal and non-temporal measures were employed as response variables in separate models, and explanatory variables were selected on the basis of whether their inclusion improved the second-order AIC (Akaike information criteria) value of the model. In addition, we evaluated absolute measures of goodness-of-fit to determine whether the included variables were indeed informative. We also estimated the variation inflation factors for each model in order to evaluate collinearity between the explanatory variables. The effect of Relative $timing_{P2}$ was evaluated using the accuracy across four consecutive episodes. Two participants were excluded from these analyses as they did not fill out the questionnaire related to the strategies used to encode and retrieve the object and episode sequences. Further, a few observations were defined as clear outliers based on visual inspection and therefore removed from the analyses. VIF = variance inflation factor. $P < 0.05$, corrected for multiple comparisons using a 5% False Discovery Rate (FDR).

Table S4. The relationship between temporal and non-temporal measures for Object scalingP1 and Episode scalingP2.

	Relative TimingP1		Object translationP1		Object scalingP1		Relative TimingP2		Episode translationP2		Episode scalingP2		Object orderO1		Episode orderO2		Object Recognition		Object association	
Predictors	*Est*	*stat*	*Est*	*stat*	*Est*	*stat*	*Est*	*stat*	*Est*	*stat*	*Est*	*stat*	*Est*	*stat*	*Est*	*stat*	*Est*	*stat*	*Est*	*stat*
(Intercept)	230.1 *	4.8	-14.1 *	-8.2	2.0 *	24.0	-2.8	-1.4	-3.6 *	-4.5	0.9 *	46.4	-1 *	-11	-6*	-15	2.6 *	6.8	-0.3*	-2.4
TranslationP11	7.9 *	61.5			0.0 *	9.9							-0 *	-3.5						
Episode translationP2	-3.1 *	-8.5			-0.0 *	-2.4	0.1 *	8.3			0.0 *	8.9	0.0 *	2.5	0.0 *	2.2				
Event orderO11	17.4 *	16	-0.2 *	-3.6	0.0	1.5	-0.1	-1.8	0.0 *	2.2					0.0	0.4	0.1 *	11	0.0	1.8
Q1 rythm	9.8	1.8																	0.0	1.5
Events duration	-2.7 *	-30	0.1 *	30.7	-0.0 *	-33			0.0	1.8	-0.0 *	-2.2	0.0 *	5.9	0.0 *	3.8	0.0 *	4.9	-0*	-2.4
Temporal variabilityP1	-115*	-31	0.4 *	2.3	0.6 *	22.8			-0.2 *	-4.1	0.0 *	5.6	0.1*	3.8	0.1	2.1				
Q7 objName	-7.5	-1.5					0.2	1.5					0.0	2.0			0.1 *	3.4	0.0 *	2.2
Event test duration	0.0	1.3	0.0 *	5.8	0.0	0.4	-0.1*	-47	0.0	2.0	-0.0 *	-7.6	-0.0	-1.5	0.0	0.7	0.0	1.3		
ObjectRecognition	3.3 *	3.1	0.1	1.4			0.1 *	3.3			-0.0	-1.8	0.1 *	12.3					0.0 *	16.3
Episode duration	-0.0 *	-8.4	0.0 *	2.2					-0.0	-1.9	0.0 *	3.9	-0.0*	-3.2	0.0	1.9				
Run nr	-10.9 *	-9.0	-0.1	-1.7			-0.7*	-15	-0.2 *	-10.9			-0.1*	-18	-0.1*	-3.8	0.2 *	27	0.0 *	11
Episode orderO2	0.7 *	2.2							0.0	2.0	0.0 *	2.2							0.0 *	2.9
Object association	7.3 *	3.0									0.0 *	2.7	0.0	2.0	0.1 *	3.1	0.2 *	16.1		
Temporal variabilityP2	3.7	1.7	-0.2 *	-2.2	0.0	1.9	-7.8 *	-111	-0.3 *	-9.7	0.0 *	4.0	0.0	1.9			-0.0 *	-2.3	0.0	1.7
Object scalingP1	-5.2 *	-6.5	0.3 *	9.4			0.0	1.6	-0.0 *	-2.8	0.0	1.5								
Episode nr in run[1st deg.]	-63.4	-0.3					-125*	-19	-98 *	-36	7.7 *	32.2			-5.8	-1.9			12**	29
Episode nr in run [2nd deg.	-459*	-2.5					-68*	-10	82.6 *	30.1	8.9 *	37.1			-91*	-29			11*	25
Episode scalingP2	11.1 *	2.7	-0.3	-1.7	0.0	1.8	3.6 *	23.9	0.5 *	8.0			0.1 *	3.7	0.2 *	2.4	-0.1 *	-2.5	0.0 *	3.0
RelativeTimingP2	0.2	1.6							0.0 *	8.1	0.0 *	24.4	-0.0	-1.9			0.0 *	3.1		
RelativeTimingP1			0.0 *	63.5	-0.0 *	-2.4	0.0	1.5	-0.0 *	-8.9	0.0 *	2.8	0.0 *	16.4	0.0 *	3.0	0.0 *	3.7	0.0 *	3.4
Q2 countedSec			0.2 *	2.5			0.3 *	3.0	0.1	1.4									0.0	2.1
Age			-0.2 *	-3.2			0.1	1.9											0.0	2.1
objPosNr [1st degree]			28.3 *	3.9	12.5 *	10.6							-2.4*	-2.7						
objPosNr [2nd degree]			86.7 *	11.7	53.9 *	45.5							4.5 *	5.1						
Q14 fix objNames			-0.2 *	-2.8			-0.1	-1.5	-0.1 *	-2.5			0.0 *	4.3						
Q12 fix seqLearn			0.3 *	3.3	0.0 *	3.1			0.1	1.8							-0.1 *	-2.7		
Q18 fix relaxed			0.2	1.7			0.3 *	2.5	-0.2 *	-2.5							-0.1	-2.1		
Q16 fix ThoughtStim			-0.1	-1.6									-0 *	-2.1						
Sex			-0.7	-1.6	-0.0	-1.7							0.1 *	2.3	0.2	1.6				
Group			0.6	1.5	-0.0	-1.8			0.6 *	2.2	0.0 *	3.2			0.3 *	2.5				
Q6 objCat					-0.0 *	-2.8														
Q8 objAss					0.0 *	2.3	-0.1	-1.4												
Q10 objSubGrp					0.0	1.5	-0.2*	-3.3					-0.0	-1.8						
Q20 OE focused							0.2	1.9							-0	-1.5			-0	-1.6
Q17 fix mindWander							-0.2	-1.9												
Q9 imgMovEnv							0.2	1.9					0.1 *	6.1	0.1 *	2.4	0.1 *	2.3	0.0 *	2.3
Q13 fix replayedSeq							0.2	1.8									0.1 *	2.5	0.0	1.7
Q21 OE mindWander													0.0 *	2.8						
Q22 objDetails													-0.0	-2.0	0.1 *	2.5	0.1 *	2.1		
Episode nr in run													-0.0 *	-7.2			0.0 *	2.3		
Q4 spatialPos															0.1	2.0				
Q15 fix compSeq															-0.1	-2.0	-0.1	-1.5		
Q5 stories																			0.0 *	5.0
Q11 objANumber																			-0*	-2.1
Q19 OE learnSeq																			-0	-1.7
**Random Effects**																				
σ^2	30014.31		50.04		1.36		40.58		6.80		0.05		0.75		8.68		0.81		0.16	
τ_{00}	23899.09 Id		4.56 Id		0.01 Id		4.87 Id		2.25 Id		0.00 Id		0.07 Id		0.51 Id		0.59 Id		0.02 Id	
ICC	0.44		0.08		0.01		0.11		0.25		0.06		0.08		0.05		0.42		0.10	
N	136 Id		136 Id		136 Id		136 Id		136 Id		138 Id		136 Id		136 Id		136 Id		136 Id	
Observations	32510		32510		32510		32510		32510		32990		32510		32510		32510		32520	
Marginal R^2/Conditional R^2	0.128 / 0.515		0.213 / 0.279		0.113 / 0.119		0.517 / 0.569		0.109 / 0.331		0.118 / 0.170		0.100 / 0.173		0.041 / 0.094		0.153 / 0.511		0.118 / 0.206	
**VIF (avg(range))**	1.09(1-2)		1.08(1-2)		1.11(1-2)		1.12(1-2)		1.1(1-2)		1.11(1-2)		1.13(1-2.1)		1.06(1-2)		1.24(1.01-1.84)		1.08(1-2)	

In these analyses, the temporal and non-temporal measures were employed as response variables in separate models, and explanatory variables were selected on the basis of whether their inclusion improved the second-order AIC (Akaike information criteria) value of the model. In addition, we evaluated absolute measures of goodness-of-fit to determine whether the included variables were indeed informative. We also estimated the variation inflation factors for each model in order to evaluate collinearity between the explanatory variables. The effect of Relative timing$_{P2}$ was evaluated using the accuracy across four consecutive episodes. Two participants were excluded from these analyses as they did not fill out the questionnaire related to the strategies used to encode and retrieve the object and episode sequences. Further, a few observations were defined as clear outliers based on visual inspection and therefore removed from the analyses. VIF = variance inflation factor. P < 0.05, corrected for multiple comparisons using a 5% False Discovery Rate (FDR).

Table S5. Activation pattern integration within episodes for $Scaling_{P1}^{Compr} > Scaling_{P1}^{Exp}$.

Brain region	Hemisphere	Cluster nr	Cluster size	t-value(max)	p-value	X	Y	Z
Visualcortex, v4	L	51	783	4	.002	-27.6	-81.4	-6
Visualcortex, v1	R	51	783	3.99	.002	8.11	-90.3	0
Lateral occipital cortex	L	51	783	3.92	.002	-27.6	-84.4	3
Visualcortex v2	L	51	783	3.81	.002	-9.73	-81.4	0
Visualcortex v2	R	51	783	3.79	.002	17	-96.3	15
Occipital pole	R	51	783	3.78	.002	28.9	-93.3	18
Lateral occipital cortex	L	51	783	3.73	.002	-42.4	-81.4	6
Occipital pole	L	51	783	3.68	.002	-36.5	-96.3	18
Lateral occipital cortex	L	51	783	3.68	.002	-57.3	-78.4	12
Occipital pole	R	51	783	3.65	.002	28.9	-87.4	6
Cerebellum	L	50	533	4.09	.002	-24.6	-30.9	-48
Cerebellum,IX	R	50	533	3.9	.002	5.14	-42.8	-42
Cerebellum,VIIIa	L	50	533	3.83	.002	-21.6	-63.6	-48
Cerebellum,CrusII	L	50	533	3.8	.002	-36.5	-63.6	-48
Temporal fusiform cortex	L	50	533	3.79	.002	-33.5	-27.9	-30
Cerebellum,VI	L	50	533	3.78	.002	-33.5	-45.7	-42
Cerebellum,VIIIb	R	50	533	3.77	.002	8.11	-42.8	-57
Cerebellum,IX	L	50	533	3.77	.002	-15.7	-45.7	-48
Cerebellum,X	R	50	533	3.74	.002	17	-42.8	-48
Cerebellum,IX	R	50	533	3.72	.002	8.11	-45.7	-30
Cerebellum,IIV	L	50	533	3.71	.002	-24.6	-30.9	-36
Cerebellum,VIIIb	L	50	533	3.66	.002	-15.7	-57.6	-60
Cerebellum,VIIIa	L	50	533	3.66	.002	-30.5	-51.7	-51
Visualcortex,v1	R	49	127	3.88	.002	20	-60.6	6
Temporal occipital fusiform cortex	R	49	127	3.75	.002	34.9	-57.6	-3
Occipital fusiform gyrus	R	49	127	3.72	.002	34.9	-63.6	-9
Visualcortex, v4	R	49	127	3.68	.002	40.8	-69.5	-12
LingualGyrus,anterior	L	48	74	3.87	.002	-6.76	-57.6	0
Cerebellum,V	R	48	74	3.77	.002	2.16	-63.6	-12
LingualGyrus,anterior	L	48	74	3.7	.002	-9.73	-66.5	-6
LingualGyrus,posterior	R	48	74	3.68	.002	5.14	-75.5	-12
Cerebellum,VI	L	47	57	3.74	.002	-21.6	-51.7	-27
Cerebellum,	L	47	57	3.73	.002	-24.6	-54.6	-36
Cingulate gyrus,posterior	R	46	51	3.88	.002	2.16	-48.7	27
Cerebellum,VIIb	R	45	46	3.83	.002	8.11	-81.4	-54
Cerebellum,VIIIa	R	45	46	3.64	.002	11.1	-63.6	-39
Insula,posterior	R	44	34	3.75	.002	34.9	-19	21
Cingulate gyrus,posterior	L	43	28	3.92	.002	-12.7	-27.9	36
Thalamus,Temporal	R	42	25	3.73	.002	14.1	-21.9	15
Orbitofrontal cortex	L	41	24	3.89	.003	-14	58.3	-25
Middle temporal gyrus	L	40	23	3.69	.003	-51.4	-42.8	-3
Thalamus,Prefrontal	R	39	17	3.68	.003	5.14	-27.9	3
Parahippocampal cortex,posterior	L	38	16	3.71	.003	-15.7	-42.8	-12
Middle temporal gyrus	R	37	16	3.74	.003	40.8	-39.8	15
Temporal fusiform cortex	L	36	14	3.8	.003	-33.5	-7.08	-51
Occipital pole	L	35	11	3.77	.003	-27.6	-96.3	30
Temporal pole	R	34	11	3.76	.003	23	22.7	-33
Perirhinal cortex	R	33	10	3.7	.004	31.9	10.8	-36
Precentral gyrus	R	32	10	3.73	.004	31.9	4.81	27
Hippocampus,posterior	L	31	9	3.7	.004	-30.5	-36.8	-15
Cerebellum,VIIIa	R	30	9	3.64	.004	31.9	-57.6	-54
Caudate,posterior	L	29	8	3.91	.004	-21.6	-27.9	18
Cuneal cortex	R	28	6	3.63	.004	20	-69.5	21
Middle temporal gyrus	R	27	5	3.68	.004	43.8	-36.8	-3
Heschl gyrus	R	25	5	3.66	.004	37.8	-27.9	9
Temporal fusiform cortex	R	24	5	3.7	.004	28.9	-16	-42
Cerebellum,	L	23	5	3.66	.004	-3.78	-36.8	-18
LingualGyrus,anterior	L	22	4	3.65	.004	-21.6	-57.6	-6
Cerebellum,VI	R	21	4	3.64	.004	14.1	-69.5	-24
Temporal pole	L	20	4	3.66	.004	-33.5	16.7	-39
Callosal cortex	L	19	4	3.67	.005	-24.6	31.6	0
Heschl gyrus	L	18	4	3.65	.005	-51.4	-19	6
Occipital pole	L	17	3	3.62	.005	-9.73	-99.2	30
Lateral occipital cortex	R	16	3	3.64	.005	28.9	-75.5	27

The activation pattern dissimilarity analysis tested whether the activation patterns for individual events within episodes became more or less similar with increasing encoded accuracy during the stimulus period. The activation pattern dissimilarity analysis was carried out using a corrected cluster mass threshold of $p = 0.05$. Only clusters that were larger than 2 voxels in 2 mm MNI (Montreal Neurological Institute) space were reported, and up to 35 local maxima were reported for each cluster. If there were several local maxima with the same name for one specific cluster, then only the local maxima with the highest t-value was kept in the table. The p-values are cluster mass corrected, with one p-value being reported for each cluster. R, right; L, left. X, Y, and Z indicates the position of the max t-value in 2 mm MNI space.

Table S6. Activation pattern separation between episodes for Episode scaling$_{P2}^{Exp}$

BrainRegion	Hemisphere	ClusterNr	ClusterSize	t-value(max)	p-value	X	Y	Z
**Stimulus**								
Precentral gyrus	R	56	2573	5.18	.001	46.8	-1.13	30
Insula,posterior	R	56	2573	4.94	.001	37.8	-19	9
Inferior temporal gyrus	R	56	2573	4.77	.001	64.6	-33.8	-21
Supramarginal gyrus	R	56	2573	4.75	.001	64.6	-21.9	27
Inferior parietal cortex,PFcm	R	56	2573	4.71	.001	46.8	-36.8	24
Middle frontal gyrus	R	56	2573	4.7	.001	31.9	7.79	45
Middle temporal gyrus	R	56	2573	4.65	.001	70.5	-27.9	-27
Temporal pole	R	56	2573	4.59	.001	52.7	19.7	-18
Brocas area	R	56	2573	4.52	.001	46.8	4.81	21
Superior temporal gyrus	R	56	2573	4.51	.001	64.6	-27.9	6
Inferior parietal cortex,Pga	R	56	2573	4.51	.001	55.7	-54.6	15
Planum temporale	R	56	2573	4.5	.001	31.9	-33.8	18
Thalamus,Premotor	R	56	2573	4.45	.001	17	-19	15
Primary motor cortex,BA4a	R	56	2573	4.44	.001	49.7	-10.1	45
Caudate,posterior	R	56	2573	4.44	.001	20	-21.9	21
Parietal operculum cortex	R	56	2573	4.4	.001	58.6	-24.9	15
Putamen,posterior	R	56	2573	4.38	.001	28.9	-4.11	6
Inferior frontal gyrus	R	56	2573	4.34	.001	58.6	16.7	30
Secondary somatosensory cortex	R	56	2573	4.3	.001	52.7	-7.08	21
Inferior parietal cortex	R	56	2573	4.29	.001	61.6	-30.9	30
Primary somatosensory cortex,BA3b	R	56	2573	4.28	.001	46.8	-16	39
Heschl gyrus	L	55	1016	5.54	.002	-48.4	-13	3
Inferior temporal gyrus	L	55	1016	5.29	.002	-54.3	-13	-45
Insula,anterior	L	55	1016	5.26	.002	-33.5	10.8	0
Thalamus,Prefrontal	L	55	1016	4.8	.002	-12.7	-4.11	6
Pallidum	L	55	1016	4.73	.002	-24.6	-13	3
Superior temporal gyrus	L	55	1016	4.52	.002	-57.3	-10.1	0
Secondary somatosensory cortex	L	55	1016	4.36	.002	-48.4	-24.9	18
Putamen,posterior	L	55	1016	4.33	.002	-33.5	-1.13	-3
Insula,posterior	L	55	1016	4.22	.002	-39.5	-10.1	-3
Central opercular cortex	L	55	1016	4.16	.002	-48.4	7.79	3
Middle temporal gyrus	L	55	1016	4.15	.002	-63.2	-4.11	-24
Caudate,anterior	L	55	1016	4.12	.002	-6.76	7.79	15
Temporal fusiform cortex	L	55	1016	4.04	.002	-42.4	-42.8	-21
Thalamus,parietal	L	55	1016	4.03	.002	-21.6	-30.9	3
Hippocampus,anterior	L	55	1016	3.88	.002	-18.6	-13	-18
Angular gyrus	L	54	472	4.75	.003	-57.3	-57.6	24
Postcentral gyrus	L	54	472	4.45	.003	-60.3	-24.9	39
Supramarginal gyrus	L	54	472	4.37	.003	-63.2	-48.7	30
Secondary somatosensory cortex	L	54	472	4.25	.003	-45.4	-30.9	24
Superior parietal cortex	L	54	472	4.25	.003	-42.4	-39.8	45
Primary somatosensory cortex,BA3aL	L	54	472	4.23	.003	-42.4	-16	36
Inferior parietal cortex	L	54	472	4.13	.003	-57.3	-30.9	45
Precentral gyrus	L	54	472	4.07	.003	-39.5	-4.11	33
Anterior intraparietal cortex	L	54	472	4.02	.003	-45.4	-39.8	36
Superior temporal gyrus	L	54	472	3.93	.003	-69.2	-39.8	27
Inferior parietal cortex	L	54	472	3.72	.003	-60.3	-16	30
Cingulate gyrus,posterior	L	53	335	4.98	.004	-12.7	-48.7	33
Premotor cortex,BA6	L	53	335	4.53	.004	-0.81	-16	60
Premotor cortex,BA6	R	53	335	4.45	.004	2.16	-13	69
Precentral gyrus	L	53	335	4.14	.004	-9.73	-21.9	45
Supplementary motor cortex	L	53	335	4.13	.004	-6.76	-1.13	69
Primary motor cortex	R	53	335	3.84	.004	8.11	-27.9	54
Primary somatosensory cortex	R	53	335	3.74	.004	17	-42.8	51
Primary motor cortex,BA4a	L	53	335	3.64	.004	-0.81	-27.9	51
Superior parietal cortex,7A	L	52	257	4.89	.004	-21.6	-51.7	63
Superior parietal cortex , 5	L	52	257	4.51	.004	-0.81	-42.8	75
Superior parietal cortex	L	52	257	4.24	.004	-33.5	-48.7	72
Primary motor cortex,BA4a	L	52	257	4.17	.004	-6.76	-42.8	81
Primary somatosensory cortex	R	52	257	3.67	.004	8.11	-45.7	66
Cerebellum,CrusII	R	51	89	4.25	.007	43.8	-63.6	-45
Cerebellum,VI	R	51	89	3.83	.007	31.9	-45.7	-42
Cerebellum,CrusI	R	51	89	3.73	.007	55.7	-66.5	-36
Primary somatosensory cortex	R	50	81	4.04	.009	34.9	-39.8	45
Anterior intra parietal cortex	R	50	81	3.88	.009	43.8	-45.7	39
Superior parietal cortex	R	50	81	3.82	.009	34.9	-45.7	39
Primary motor cortex	R	50	81	3.72	.009	34.9	-21.9	48
Cingulate gyrus,anterior	R	49	70	4.32	.009	8.11	1.84	39
Cingulate gyrus,anterior	L	49	70	3.93	.009	-6.76	16.7	36
Occipital fusiform gyrus	L	48	56	4.47	.011	-30.5	-66.5	-3
LingualGyrus,anterior	L	48	56	4.11	.011	-27.6	-51.7	-6
Parahippocampal gyrus	L	48	56	3.82	.011	-24.6	-42.8	-3
Cerebellum,CrusII	L	47	50	4.63	.012	-36.5	-45.7	-45
Cerebellum,CrusI	L	47	50	4.22	.012	-30.5	-54.6	-42
Cerebellum,VI	L	46	41	4.37	.014	-21.6	-51.7	-21
Temporal fusiform cortex	L	46	41	3.64	.014	-24.6	-39.8	-21
Precuneous cortex	L	45	40	4.16	.014	-0.81	-54.6	15
Cingulate gyrus,posterior	R	45	40	3.93	.014	8.11	-48.7	6
Precuneous cortex	R	44	39	4.11	.014	17	-54.6	21
Cerebellum,IX	R	43	39	4.43	.014	14.1	-45.7	-39
Lateral occipital cortex	R	42	38	3.99	.015	25.9	-63.6	42
Superior parietal cortex	R	42	38	3.73	.015	34.9	-54.6	39
Cerebellum,VIIb	L	41	33	4.32	.017	-6.76	-72.5	-45
Cerebellum,VIIIa	L	41	33	4.04	.017	-12.7	-63.6	-39
Precuneous cortex	R	40	33	4.16	.017	14.1	-63.6	42
Superior parietal cortex,7P	R	40	33	4.02	.017	11.1	-69.5	54
Occipital pole	R	39	30	4.33	.018	5.14	-102	-3
Occipital pole	L	39	30	4.11	.018	-0.81	-96.3	-12
Angular gyrus	R	38	29	4.01	.019	64.6	-54.6	33
Visualcortex, v1	L	37	29	4.29	.018	-12.7	-87.4	3
Occipital pole	L	37	29	3.62	.018	-21.6	-93.3	-12
Lateral occipital cortex	L	36	27	4.11	.02	-33.5	-81.4	9
Entorhinal cortex,intermediate,lateral	L	35	26	4.18	.02	-24.6	-10.1	-30
Temporal fusiform cortex	L	35	26	3.92	.02	-36.5	-10.1	-36
Cerebellum,IIV	R	34	24	4.39	.021	2.16	-42.8	-18
Cerebellum,V	R	34	24	4.08	.021	11.1	-48.7	-12
Intracalcarine cortex	L	33	21	4.11	.024	-27.6	-63.6	9
Precuneous cortex	L	33	21	3.94	.024	-21.6	-60.6	12
Cingulate gyrus,posterior	R	32	18	4.18	.025	8.11	-36.8	27

Frontal pole	R	31	18	4	.026	28.9	43.5	15
Supplementary motor cortex	R	30	16	4.03	.028	8.11	4.81	54
Orbitofrontal cortex	R	29	16	4.25	.027	17	19.7	-24
Cerebellum,CrusI	R	28	14	4.05	.031	11.1	-75.5	-24
Cerebellum,VermisVIIb	R	28	14	3.75	.031	5.14	-66.5	-30
Cingulate gyrus,posterior	L	27	14	4.49	.029	-6.76	-19	27
Superior frontal gyrus	L	26	13	4.13	.032	-9.73	28.6	42
Cerebellum,CrusII	L	25	13	3.88	.032	-27.6	-66.5	-42
Intracalcarine cortex	L	24	13	3.96	.032	-9.73	-72.5	12
Premotor cortex,BA6	L	23	12	3.8	.034	-36.5	-19	63
Primary motor cortex,BA4p	L	22	11	3.9	.035	-42.4	-16	45
Primary somatosensory cortex,BA3b	L	22	11	3.79	.035	-45.4	-19	51
Cingulate gyrus,anterior	L	21	10	3.94	.036	-6.76	1.84	39
Paracingulate gyrus	L	20	10	3.92	.037	-0.81	37.5	36
Lateral occipital cortex	L	19	9	3.84	.04	-21.6	-66.5	48
Middle frontal gyrus	L	18	9	3.75	.04	-51.4	19.7	30
Inferior frontal gyrus	L	18	9	3.73	.04	-42.4	16.7	24
Caudate,posterior	L	17	9	3.98	.04	-18.6	-13	24
Cerebellum,CrusI	L	16	9	3.89	.039	-54.3	-63.6	-33
Temporal occipital fusiform cortex	R	15	9	3.81	.04	28.9	-45.7	-15
Superior parietal cortex	R	14	8	4.43	.04	23	-54.6	51
Cingulate gyrus,anterior	L	13	8	3.8	.041	-6.76	37.5	3
Caudate,anterior	R	12	8	3.76	.042	11.1	13.7	0
Inferior parietal cortex	L	11	7	3.79	.044	-42.4	-51.7	54
Cerebellum,VIIIa	R	10	7	3.86	.044	8.11	-66.5	-42
LingualGyrus,posterior	L	9	7	3.93	.044	-0.81	-75.5	-3
Cingulate gyrus,anterior	R	8	7	3.83	.045	8.11	28.6	15
Entorhinal cortex,anterior,lateral	R	7	7	4.09	.043	23	-4.11	-45
Middle frontal gyrus	L	6	7	4.35	.042	-33.5	28.6	21
Insula,anterior	R	4	6	3.99	.049	40.8	10.8	-6
Putamen,anterior	R	4	6	3.65	.049	34.9	4.81	-3
LingualGyrus,anterior	R	3	6	4.19	.046	23	-60.6	0
Orbitofrontal cortex	R	2	6	3.77	.048	28.9	22.7	-9
Cerebellum,CrusII	R	1	6	3.75	.049	2.16	-84.4	-39
**Cross fixation**								
Supramarginal gyrus	R	10	24	3.94	.024	70.5	-21.9	24
Postcentral gyrus	L	9	20	4.52	.026	-60.3	-21.9	39
Brocas area	R	8	15	3.85	.033	58.6	10.8	3
Central opercular cortex	R	8	15	3.77	.033	49.7	4.81	9
Inferior frontal gyrus	R	7	13	3.79	.035	55.7	16.7	21
Postcentral gyrus	R	6	13	3.89	.035	49.7	-19	66
Superior temporal gyrus	L	5	10	3.87	.041	-60.3	-16	-6
Brocas area	R	4	9	3.84	.042	43.8	1.84	30
Middle temporal gyrus	R	3	7	4.03	.049	73.5	-27.9	-9
Inferior frontal gyrus	R	2	7	3.85	.048	55.7	34.5	12
Supramarginal gyrus	R	1	7	3.78	.049	61.6	-24.9	36
**Odd-even**								
na								
**Event test planning**								
na								
**Event test execution**								
na								

The activation pattern dissimilarity analysis tested whether the activation patterns between episodes became more or less similar with increasing encoded accuracy. The activation pattern dissimilarity analysis was carried out using a corrected cluster mass threshold of p = 0.05. Only clusters that were larger than 2 voxels in 2 mm MNI (Montreal Neurological Institute) space were reported, and up to 35 local maxima were reported for each cluster. If there were several local maxima with the same name for one specific cluster, then only the local maxima with the highest t-value was kept in the table. The p-values are cluster mass corrected, with one p-value being reported for each cluster. R, right; L, left. X, Y, and Z indicates the position of the max t-value in 2 mm MNI space.
